# Exploring the Interaction of New Pyridoquinazoline Derivatives with G-Quadruplex in the c-MYC Promoter Region

**DOI:** 10.3390/ijms241814346

**Published:** 2023-09-20

**Authors:** Salvatore Princiotto, Maria Karelou, Rachel Ioannidi, Giovanni Luca Beretta, Nadia Zaffaroni, Roberto Artali, Ioannis K. Kostakis, Stefania Mazzini, Sabrina Dallavalle

**Affiliations:** 1 Department of Food, Environmental and Nutritional Sciences (DeFENS), Università degli Studi di Milano, Via Celoria 2, 20133 Milan, Italy; salvatore.princiotto@unimi.it (S.P.); sabrina.dallavalle@unimi.it (S.D.); 2 Department of Pharmacy, Division of Pharmaceutical Chemistry, National and Kapodistrian University of Athens, Panepistimiopolis, Zografou, 15771 Athens, Greece; marykar@pharm.uoa.gr (M.K.); rioannidi@pharm.uoa.gr (R.I.); 3 Molecular Pharmacology Unit, Department of Experimental Oncology, Fondazione IRCCS Istituto Nazionale Tumori, Via Amadeo 42, 20133 Milan, Italy; giovanni.beretta@istitutotumori.mi.it (G.L.B.); nadia.zaffaroni@istitutotumori.mi.it (N.Z.); 4 Scientia Advice di Roberto Artali, 20832 Desio, Italy; roberto.artali@scientia-advice.com

**Keywords:** c-MYC, G-quadruplex, BMH21, antiproliferative activity, NMR spectroscopy, molecular modeling

## Abstract

Novel amino-substituted pyridoquinazolinone derivatives have been designed and synthesized as potential c-MYC G-quadruplex (G4) ligands, employing an efficient methodology. All the new compounds exhibited moderate to good antiproliferative activity against the human osteosarcoma U2OS cell line. NMR and docking experiments revealed that the recently synthesized compounds interact with the Pu22 G-quadruplex in the c-MYC promoter region, establishing a 2:1 complex, with each molecule positioned over the tetrads at the 3′- and 5′-ends.

## 1. Introduction

Cancer is one of the most widespread and aggressive diseases in the world, whose treatment demands innovative approaches [1,2,3]. A significant challenge with conventional antitumor agents lies in their strong toxicity, due to their poor selectivity [4]. Cancer includes a broad group of disorders resulting from mutations, deletions, or amplifications in oncogenes encoding for regulatory proteins [5]. Among these oncogenes is c-MYC, responsible for encoding a transcription factor that controls cell-cycle progression, cell differentiation, apoptosis, DNA replication, and mRNA maturation [6]. Under normal conditions, c-MYC expression is highly regulated. However, in many tumor cells, it is amplified, leading to cancer development [7]. c-MYC transcriptional regulation is complex and involves various elements, including several DNA-binding proteins. The recognition region for the activation of c-MYC transcription is a GC-rich sequence called Pu27, which can form intramolecular G-quadruplex (G4) structures that are able to silence c-MYC transcription [8,9].

G-quadruplexes are four-stranded structures formed from DNA or RNA sequences comprising a stack of multiple guanine (G) tetrads. Each G-tetrad (G4) is a square planar arrangement of four guanines stabilized by Hoogsteen hydrogen bonds [10,11]. G4s are not randomly distributed through the genome, being clustered in key regulatory sites such as telomeres and promoter regions of proto-oncogenes [12]. A link between the ligand-mediated stabilization of G-quadruplexes in gene promoters and transcriptional regulation has been proposed for several oncogenes [13]. Therefore, quadruplex structures are considered attractive molecular targets for cancer therapeutics with novel mechanisms of action. As the overexpression of the c-MYC oncogene is one of the most common aberrations of a wide range of human tumors, the stabilization of G-quadruplex by small molecules in the c-MYC promoter has been proposed as a promising antitumor strategy [14]. Over the past decades, numerous G4 ligands with diverse chemotypes and structures have been investigated as potential c-MYC binding molecules [6]. These compounds typically consist of a polyaromatic core with positively charged side chains, which can interact with the G4 tetrads, their phosphate backbone, and water molecules within the grooves.

**Figure 1:**
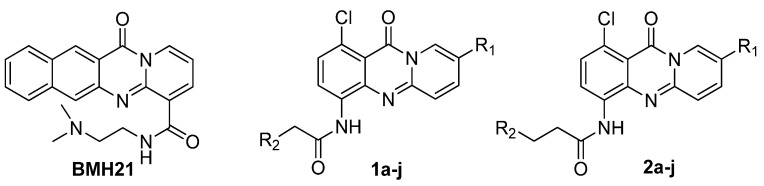
Structure of BMH21 and general structure of synthesized 11*H* pyrido[2,1-*b*]quinazolin-11-ones **1a**–**j** and **2a**–**j**.

Our research group has long been committed to the investigation of new chemotypes interacting with the G-quadruplex present in the c-MYC promoter. Recently, we provided evidence that compound BMH21, a novel intercalating agent that selectively binds to GC-rich DNA sequences [15,16], effectively binds to the tested G-quadruplex (Figure 1) [17]. BMH21 consists of a four-member heterocyclic ring system, bearing a *N*,*N*-dimethylamino carboxamide arm at position four. Docking and molecular pharmacology studies support the beneficial role of the heterocyclic ring system and the basic side chain in enhancing activity [18]. Motivated by the intriguing activity of BMH21 and its poor synthetic versatility, we decided to further investigate this scaffold. For this purpose, we synthesized some structurally related 11*H*-pyrido[2,1-*b*]quinazolin-11-ones (compounds **1a**–**j** and **2a**–**j**, Figure 1), aiming to identify the optimal structural requirements for the biological activity. To the best of our knowledge, this novel scaffold has never been investigated as a G4 stabilizer. Building on our experience, we designed and synthesized a series of derivatives following a ring contraction strategy. In these compounds, the basic side chain was repositioned onto the phenyl group of the quinazolinone ring (rather than the pyridine group, as in BMH21). Additionally, we positioned the carbonyl group between the amino side chain and the chromophore to gain a better insight into the influence of the flexibility and spatial arrangement of the amide side chain on the biological activity. Furthermore, we modulated the aminoalkyl side chain to identify the optimal moiety for an interaction with the phosphate backbone, resulting in the preparation of various analogs with different aminoalkyl chains.

The antiproliferative activity and SAR of these compounds were evaluated. An investigation of the binding of a selected compound to the c-MYC G-quadruplex oncogene was undertaken by NMR and molecular modeling.

## 2. Results and Discussion

### 2.1. Chemistry

**Scheme 1:**
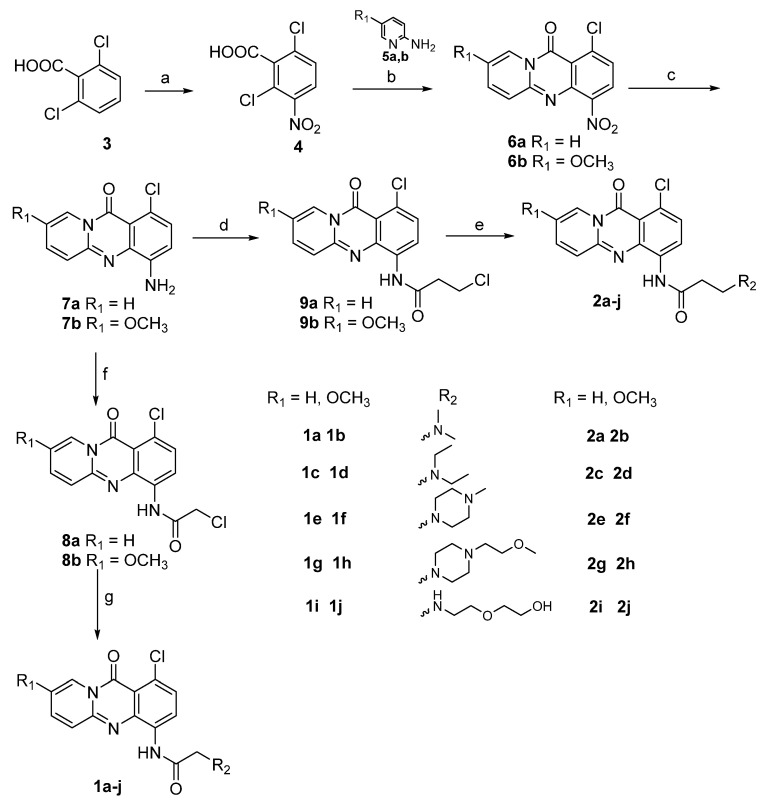
*Reagents and conditions:* (a) Fuming HNO_3_, concentrated H_2_SO_4_, rt, 45 min; (b) NaH, dry DMF, 50 °C, 24 h; (c) hydrazine hydrate, Ni-Raney, MeOH, rt, 15 min; (d) ClCOCH_2_CH_2_Cl, Na_2_CO_3_, THF, rt, 15 min; (e) suitable amine, EtOH abs., reflux, 24 h; (f) ClCOCH_2_Cl, Na_2_CO_3_, THF, rt, 15 min; (g) corresponding amine, EtOH abs., reflux, 24 h.

For the synthesis of the target compounds, we have used as starting material 2,6-dichlorobenzoic acid (Scheme 1), which was converted to the nitro compound **4** after treatment with fuming HNO_3_ in concentrated H_2_SO_4_. Reaction of this compound with 2-aminopyridines **5a**,**b** in the presence of NaH, afforded compounds **6a**,**b**, respectively. The nitro group was then easily reduced by hydrazine hydrate and Ni-Raney catalyst to afford the amino derivatives **7a**,**b**, which were readily converted to the corresponding amides **8a**,**b** and **9a**,**b**, by reaction with chloroacetyl chloride or 3-chloropropionyl chloride, respectively. Reaction of these amides with the suitable amines resulted in the target compounds **1a**–**j** and **2a**–**j**.

### 2.2. Antiproliferative Activity Evaluation

The in vitro antiproliferative activity of the new compounds was evaluated in the established model of U2OS osteosarcoma cell line (Table 1). Most of the new compounds showed interesting antiproliferative activity against the tested cell line, with IC_50_ values varying within the range of 5.9–47.33 μΜ. In general, all the compounds were less active than BMH21; however, some of them (compounds **2a**, **2h**, **2c**, **2f,** and **2d)** exhibited cytotoxicity comparable to that of RHPS4, used as a reference compound. Compounds **2j**, **2i,** and **2b** were about one and half- to two-fold more active than RHPS4 (Table 1).

In more detail, the alkylaminoacetamido substituted compounds showed very modest activity (**1a** and **1b**) or low solubility (**1e**, **1g**, **1d**, **1f** and **1h**). The best compounds of this first series were **1c**, **1i,** and **1j** (IC_50_ 34, 35, and 14 μM, respectively). Elongating the side chain by introducing a three-substituted propanoyl group in place of the acetyl residue consistently increased the activity (**2a, c, e, g, i** vs. **1a, c, e, g, i**). The introduction of a methoxy group on C-8 did not increase the activity of the acetamido-substituted derivatives, except for compound **1j** (IC_50_ 14.3 vs. 35 μM of **1i**). Conversely, the series of compounds containing the alkylaminopropanamido side chain retained (compound **2d** vs. **2c**) or increased (**2b**, IC_50_ 5.9 vs. 11 μM of **2a**; **2f,** IC_50_ 10 vs. 26 μM of **2e**; **2h**, IC_50_ 17.5 vs. 47 μM of **2g** and **2j**, IC_50_ 8 vs. 6.4 μM of **2i**) the antiproliferative potency after the introduction of the methoxy group.

### 2.3. NMR Studies

Compound **2i** was selected as a suitable training molecule to probe the binding to G4 of c-MYC oncogene by NMR spectroscopy. The purine-rich strand of the nuclease hypersensitive element (NHE) III1 sequence of the c-MYC promoter, which controls 80–90% of the c-MYC transcription is a 27 nt segment (MYCPu27), containing five consecutive runs of guanines. Pu22 is a 22 mer sequence of MYCPu27, mainly responsible for the c-MYC transcriptional activity [19].

**Figure 2:**
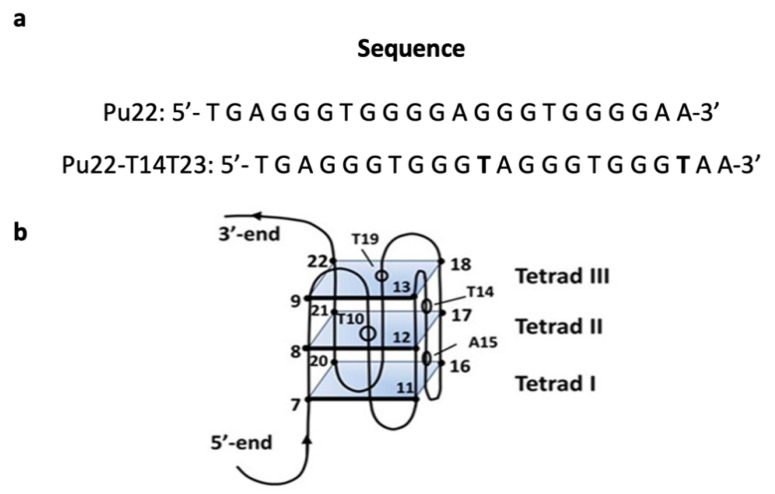
(**a**) Nucleotide sequence of Pu22 and mutated Pu22-T14T23; (**b**) rearrangement of Pu22-T14T23 in parallel G-tetrads.

Specifically, the experiments were performed with Pu22-T14T23, which is a sequence with two mutations at positions 14 and 23 of c-MYC Pu22 with respect to the native sequence (Figure 2) [20].

Several authors have used this modified oligonucleotide to study the binding properties of G-quadruplex binding drugs by NMR, as the wild sequence gave poorly resolved proton NMR spectra [17,20]. The mutations allow for the formation of a single monomeric intramolecular parallel G-quadruplex structure similar to the native form. Twelve narrow and well-resolved imino protons are present in the ^1^H NMR spectrum, which are consistent with the formation of G-quadruplex with three G-quartets.

**Figure 3:**
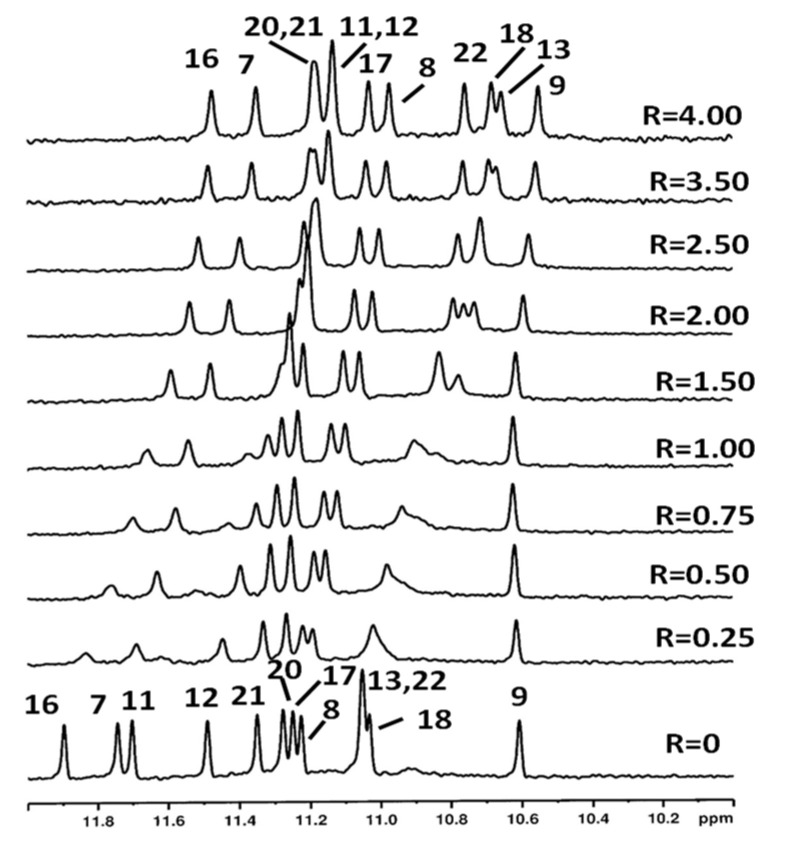
Imino protons region of the ^1^H NMR titration spectra of Pu22-T14T23 with **2i** at 25 °C in H_2_O/D_2_O (9:1), 70 mM KCl, 25 mM potassium phosphate buffer (pH 6.9).

**Figure 4:**
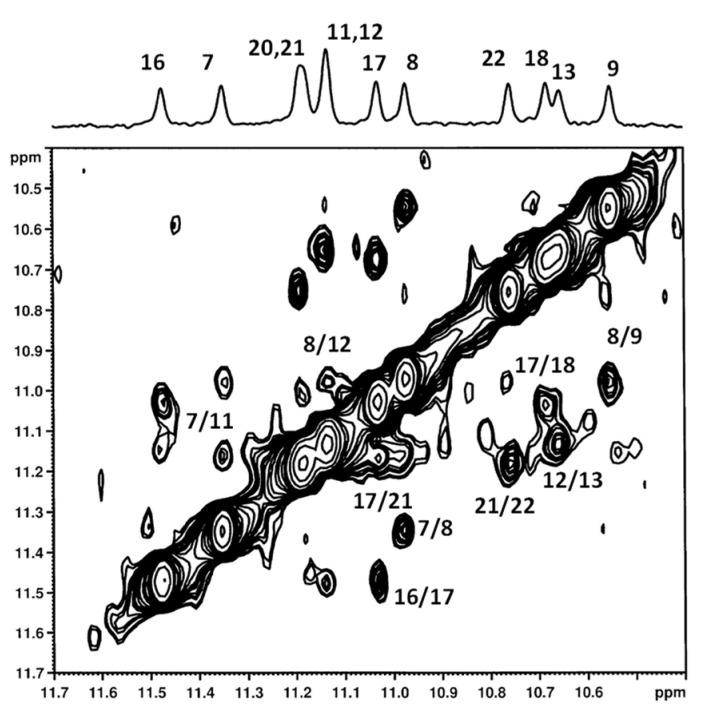
Imino protons region of 2D NOESY spectra of Pu22-T14T23 G-quadruplex with **2i** at 25 °C, mix = 350 ms in H_2_O/D_2_O (9:1), 70 mM KCl, 25 mM potassium phosphate buffer (pH = 6.9), and R = 4.0.

**Figure 5:**
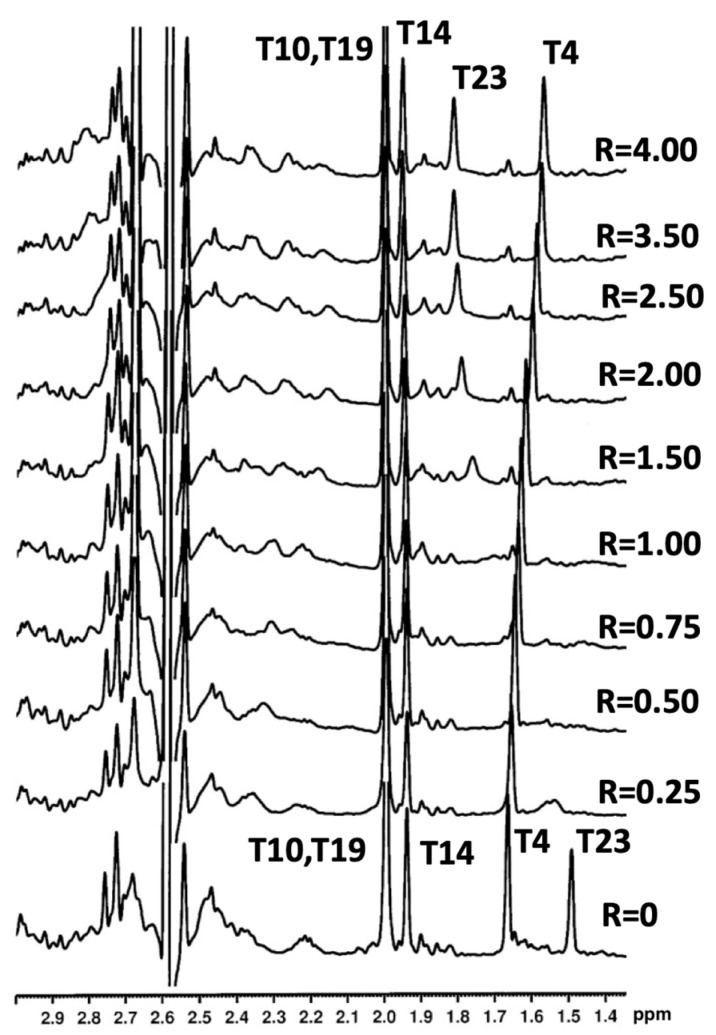
Up-field region of the ^1^H NMR titration spectra of Pu22-T14T23 with **2i** at 25 °C in H_2_O/D_2_O (9:1), 70 mM KCl, 25 mM potassium phosphate buffer (pH = 6.9).

Increasing amounts of **2i** were added to the oligonucleotide solution. The assignment of the imino NH resonances of the guanines in the complex was performed from the 1D titration spectra and by sequential NH–NH NOE (nuclear Overhauser effect) interactions by NOESY (nuclear Overhauser effect spectroscopy) experiments (Figure 3 and Figure 4). Consequently, the assignments of the aromatic protons in the complex were possible by observing the inter-residue NOE interactions with the NH imino protons, which define the patterns of the three tetrads for both G-quadruplexes. The identification of the cytidine aromatic protons was based on TOCSY (total correlation spectroscopy) experiments, as the vicinal H6 and H5 protons displayed very strong signals. The thymine protons were identified through the methyl resonances, lying up-field (Figure 5). Selected chemical shifts for the considered complexes are reported in Table 2. The protons of **2i** alone and in the complex with Pu22-T14T23 were assigned and reported in Table 3.

The NH imino protons remained in a region ranging from 10.5 to 12.0 ppm, indicating that the G-quadruplex structure was conserved after the interaction with the ligand. The NH signals in **2i**/Pu22-T14T23 complex remained quite sharp with the exception of the imino protons belonging to 3′ (G13, G18, and G22) and 5′ (G11 and G16) quartets at R = 1.0. All the signals became sharp for R > 1.0 (Figure 3). A comparison of NH chemical shifts showed that **2i** induced significant chemical shift variations (G22, G18, and G13 NH, Δδ = −0.27/−0.32/−0.38 ppm; G7, G11, G16 NH, Δδ from −0.34 to −0.56 ppm) at 3′-end and at 5′-end. Additionally, the NH of the internal tetrad showed an up-field shift, particularly evident for G12 (Δδ = −0.35 ppm) (Table 2).

The chemical shift variations suggested that **2i** could be located over the 3′-end or over the 5′-end tetrad. The deshielding of H8 A6 proton indicated that the unit at 5′-end was pushed away from the lower tetrad, being no more stacked with G7 unit. At the 3′-end, T23 unit showed a down-field shift, evidencing that a conformational change occurred (Δδ = +0.32 ppm). A24 was no more stacked by the T23:A25 base pair, and A25 was no more folded over the G9 aromatic moiety as it resulted for the free nucleotide, in agreement with the low-field shift observed for A24 (Δδ = +0.35 ppm) and A25 (Δδ = +0.10 ppm) protons.

**Figure 6:**
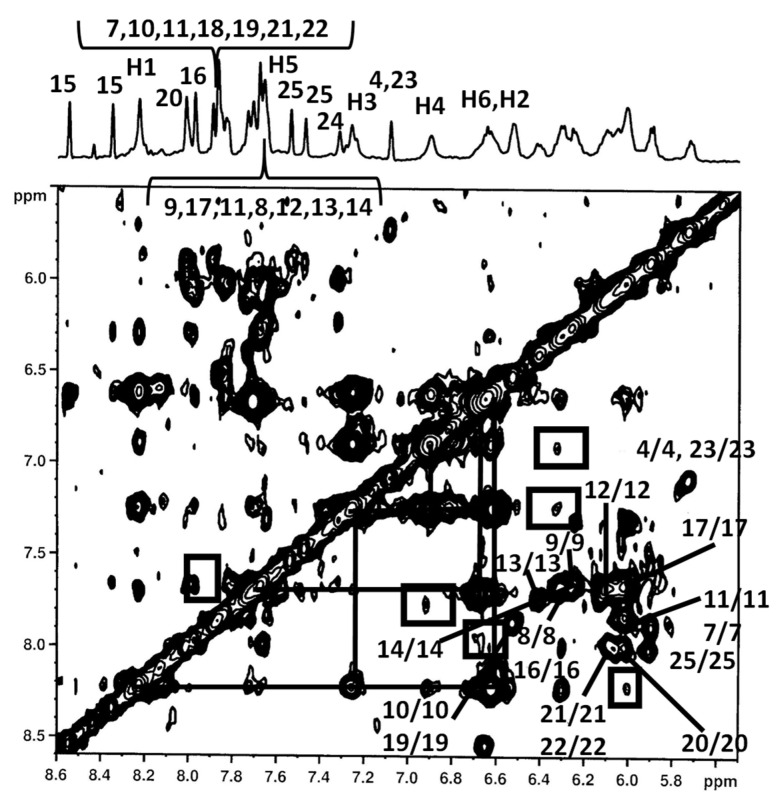
Aromatic and anomeric protons region of 2D NOESY spectra of Pu22-T14T23 G-quadruplex with **2i** at 25 °C, mix = 350 ms in H_2_O/D_2_O (9:1), 70 mM KCl, 25 mM potassium phosphate buffer (pH = 6.9), and R = 4.0. The lines indicate the **2i** aromatic protons and the boxes indicate the intermolecular interactions between Pu22-T14T23 and **2i**.

To implement the information provided by the 1D NMR studies, 2D NOESY experiments were carried out at R ratio equal to 2 and 4. The intermolecular NOE interactions (Table 4) were in agreement with the Δδ values observed and confirmed that **2i** binds to the Pu22 quadruplex forming a 2:1 complex, where one molecule is positioned over tetrad I at 5′-end and a second molecule over tetrad III at 3′-end. NOE interactions were found between the aromatic protons of the ligand and the G7, G11, and G16 units at the 5′-end and with the G13, G18, G22, and G9 units at the 3′-end (Figure 6). Ambiguities in the intermolecular interactions due to the overlapping of chemical shifts of some aromatic and NH protons of the G-quadruplex were elucidated by molecular modeling studies, which allowed for the discrimination of the position of **2i** in the binding site (see below).

In addition, DOSY (diffusion ordered spectroscopy) experiments performed on the complex displayed a coefficient diffusion value of −9.897 m^2^s^−1^ that corresponds to the formation of 2:1 complex (molecular weight = 7872).

### 2.4. Molecular Modeling Studies

**Figure 7:**
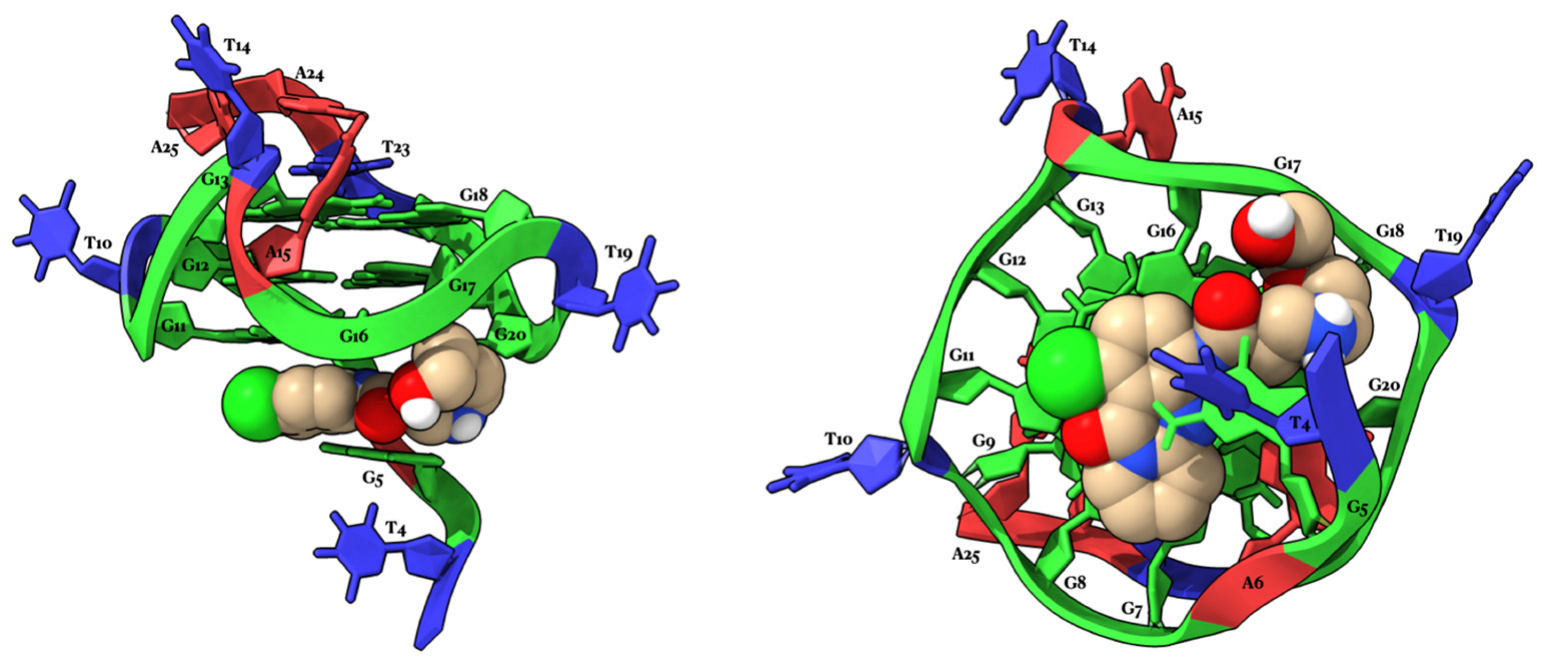
Side (**left**) and top (**right**) views of the **2i** complex with the *c-MYC* G-quadruplex sequence at the 5′-end. The aromatic moiety of the ligand is located near the center of the quadruplex, while the side chain fits into the groove between G17 and G20. The nucleotides are shown as slabs and filled sugars: adenine in red, guanine in green, and thymine in blue. Labels are added to highlight key nucleotides. The ligand molecules are rendered in CPK and colored according to the atom types. In both views, only polar hydrogens are shown.

The results of the molecular modeling study at the 5’-end showed that **2i** is perfectly inserted in the cleft created between G5 and the G7-G11-G16-G2 tetrad. The aromatic moiety of the ligand was located near the center of the quadruplex, allowing it to interact effectively with G7, G11, and G16 bases via π–π stacking interactions (Figure 7). The side chain of **2i** is instead oriented towards the area located between G17 and G20, with the amino nitrogen making an electrostatic interaction with OP2 of G20. The same charged nitrogen atom makes a hydrogen bond with G5N7 (2.69 Å), while the oxygen atom of the -CH_2_OCH_2_- group interacts with G16H4, giving rise to a further hydrogen bond at 1.97 Å.

**Figure 8:**
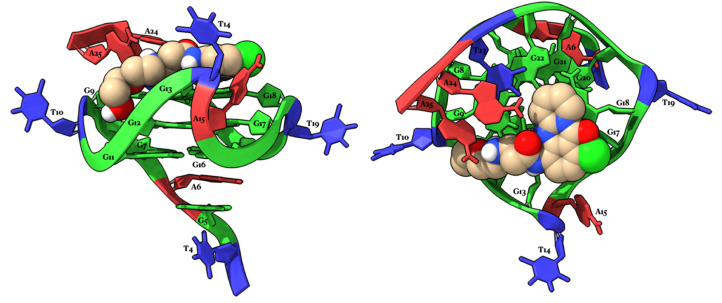
Side (**left**) and top (**right**) views of the **2i** complexes at 3′-end with the *c-MYC* G-quadruplex sequence. The ligand was drifted with respect to the center of the quadruplex, towards the G13-G18 base pair. The side chain was oriented towards the exterior of the quadruplex, in the area between G9 and G12.The nucleotides are shown as slabs and filled sugars: adenine in red, guanine in green, and thymine in blue. Labels are added to highlight key nucleotides. The ligand molecules are rendered in CPK and colored according to the atoms and only polar hydrogens are shown.

Analysis of the complex at the 3′-end showed that **2i** had the aromatic portion displaced from the center of the G9-G13-G18-G22 tetrad (Figure 8). Consequently, the only π–π stacking interactions detected were those involving the G13 and G18 bases. Again, the ligand side chain pointed towards the area located between G9 and G12. The amino nitrogen of **2i** formed a hydrogen bond with the N1 of A25 (2.18 Å), while the oxygen of -CH_2_OCH_2_- interacted with the group of G9 (2.38 Å). The side chain interaction pattern was completed by an additional hydrogen bond between -CH_2_OH and G12OP2 (2.09 Å).

## 3. Materials and Methods

### 3.1. General Experimental Procedures

All commercially available reagents and solvents were purchased from Alfa Aesar (Ward Hill, Massachusetts, MA, USA) and used without any further purification. Melting points were determined on Büchi apparatus and were uncorrected. 1D (^1^H NMR, ^13^C NMR) and 2D (COSY, NOESY, HMBC, HSQC-DEPT135) spectra were carried out on 400 or 600 MHz Bruker spectrometers, respectively Avance™ DRX and III instruments (Bruker BioSpin GmbH, Rheinstetten, Germany). Chemical shifts (δ) are expressed in ppm while coupling constants (*J*) in Hz. The multiplicity of vertices is expressed as s (singlet), d (doublet), t (triplet), q (quartet), dd (doublet of doublets), and m (multiple). Flash chromatography was performed on Merck silica gel (40–63 μm) with the indicated solvent system using gradients of increasing polarity in most cases (Merck KGaA, Darmstadt, Germany). The reactions were monitored by analytical thin-layer chromatography on pre-coated silica gel 60 F_254_ TLC plates, 0.25 mm layer thickness (Merck KGaA, Darmstadt, Germany). Mass spectra were recorded on a UPLC Triple TOF-MS: Acquity UPLC (Waters, Milford, MA, USA), Triple TOF-MS 5600+ (AB Sciex LLC, Framingham, MA, USA).

### 3.2. Synthesis

#### 3.2.1. Synthesis of 2,6-Dichloro-3-nitrobenzoic Acid (**4**)

Fuming HNO_3_ (3 mL, 71.42 mmol) was added dropwise to a suspension 2,6-dichlorobenzoic acid (10 g, 52.63 mmol) in concentrated H_2_SO_4_ (30 mL) at 0 °C, and the resulting mixture was stirred at room temperature for 45 min. After completion of the reaction, the mixture was poured into ice and the precipitate was filtered, washed with cold water (30 mL), and dried over P_2_O_5_ to afford 12.3 g of the title compound **4**, practically pure, which was used for the next step of the synthetic procedure without any further purification. Yield: 99%. Mp.: 143–144 °C (H_2_O). ^1^H NMR (400 MHz, CDCl_3_) δ (ppm) 7.92 (d, *J* = 8.8 Hz, 1H, H-4), 7.55 (d, *J* = 8.8, 1H, H-5), 6.84 (brs, 1H, OH). ^13^C NMR (101 MHz, CDCl_3_) δ (ppm) 166.6 (CO), 146.6 (C-3), 135.9 (C-2), 135.4 (C-1), 128.9 (C-6), 126.9 (C-5), 125.5 (C-4).

#### 3.2.2. Synthesis of 1-Chloro-4-nitro-11*H*-pyrido[2,1-*b*]quinazolin-11-one (**6a**) [21]

To a solution of 2-aminopyridine (600 mg, 6.38 mmol) in dry DMF (dimethylformamide) (40 mL) at 0 °C, ΝaH (60% in mineral oil) (2.5 g, 62.5 mmol) was added portion-wise and the resulting suspension was stirred under argon, at room temperature, for 30 min. The suspension was then cooled again to 0 °C, compound **4** (1.5 g, 6.38 mmol) was added, and the reaction suspension was stirred at 50 °C for 24 h. Upon cooling, the mixture was carefully poured into ice water and acidified with HCl 9% (pH ≃ 3). The resulting precipitate was filtered, washed with cold water (30 mL), and dried over P_2_O_5_ to afford 1.49 g of the title compound **6a**, as a yellow solid. Yield: 85%. Mp.: 154–156 °C (EtOAc). ^1^H NMR (600 MHz, DMSO-*d*6) δ (ppm) 8.84 (d, *J* = 7.1 Hz, 1H, H-9), 8.26 (d, *J* = 8.3 Hz, 1H, H-3), 7.88 (td, *J* = 8.4, 1.6 Hz, 1H, H-7), 7.58 (d, *J* = 8.4 Hz, 1H, H-2), 7.51 (d, *J* = 9.0 Hz, 1H, H-6), 7.20 (td, *J* = 8.4, 1.6 Hz, 1H, H-8). ^13^C NMR (151 MHz, DMSO-*d*_6_) δ (ppm) 155.5 (*C*O), 149.1 (C-5a), 145.0 (C-4a), 142.2 (C-1), 138.2 (C-7), 136.6 (C-4), 127.7 (C-3), 127.2 (C-8), 125.6 (C-6), 125.0 (C-2), 114.6 (C-11a), 114.1 (C-9).

#### 3.2.3. Synthesis of 1-Chloro-8-methoxy-4-nitro-11*H*-pyrido[2,1-*b*]quinazolin-11-one (**6b**)

The title compound was synthesized by an analogous procedure as described for the preparation of **6a**, starting from 2-amino-5-methoxypyridine. Yield: 85%. Mp.: 248–250 °C (EtOAc). ^1^H NMR (600 MHz, DMSO-*d*_6_) δ (ppm) 8.34 (d, *J* = 2.5 Hz, 1H, H-9), 8.27 (d, *J* = 8.3 Hz, 1H, H-3), 7.77 (dd, *J* = 7.07, 2.65 Hz, 1H, H-7), 7.61 (d, *J* = 8.3 Hz, 1H, H-2), 7.56 (d, *J* = 9.7 Hz, 1H, H-6), 3.93 (s, 3H, OC*H*_3_). ^13^C NMR (151 MHz, DMSO-*d*_6_) δ (ppm) 155.0 (*C*O), 149.3 (C-8), 146.6 (C-5a), 145.1 (C-4a), 141.6 (C-1), 136.4 (C-4), 133.7 (C-7), 127.3 (C-3), 126.9 (C-6), 125.2 (C-2), 113.2 (C-11a), 106.3 (C-9), 56.4 (OC*H*_3_).

#### 3.2.4. Synthesis of 4-Amino-1-chloro-11*H*-pyrido[2,1-*b*]quinazolin-11-one (**7a**)

To a solution of compound **6a** (1.2 g, 4.36 mmol) in a mixture of dry THF (20 mL) and dry MeOH (10 mL), hydrazine hydrate 98% (1.5 mL, 30.5 mmol) and Ni-Raney (50 mg) were added, and the resulting suspension was stirred for 1 h, at room temperature. After completion of the reaction, the mixture was filtered through a Celite pad and the filtrate was evaporated to dryness. The residue was purified by flash chromatography (silica gel), using a mixture of c-Hex/DCM (dichloromethane) as the eluent (2:1 to 1:1), to provide compound **7a** (540 mg) as a yellow solid. Yield: 56%. Mp.: >230 °C (EtOAc). ^1^H NMR (400 MHz, CDCl_3_) δ (ppm) 8.87 (d, *J* = 7.4 Hz, 1H, H-9), 7.61–7.48 (m, 2H, H-6, H-7), 7.27 (d, *J* = 8.4 Hz, 1H, H-2), 6.97 (d, *J* = 8.3 Hz, 1H, H-3), 6.98 (t, *J* = 6.3 Hz, 1H, H-8). ^13^C NMR (101 MHz, CDCl_3_) δ (ppm) 156.9 (*C*O), 146.6 (C-5a), 141.4 (C-4a), 134.6 (C-7), 128.0 (C-2), 126.7 (C-6, C-9), 125.8 (C-1), 120.8 (C-3), 115. 6 (C-4), 113.2 (C-8), 113.0 (C-10a).

#### 3.2.5. Synthesis of 4-Amino-1-chloro-8-methoxy-11*H*-pyrido[2,1-*b*]quinazolin-11-one (**7b**)

The title compound was synthesized by an analogous procedure as described for the preparation of **7a**, starting from **6b**. Yield: 68%. Mp.: >230 °C (EtOAc). ^1^H NMR (400 MHz, CDCl_3_) δ (ppm) 8.40 (d, *J* = 2.7 Hz, 1H, H-9), 7.44 (dd, *J* = 9.8, 0.7 Hz, 1H, H-7), 7.34 (d, *J* = 9.8 Hz, 1H, H-6), 7.28 (d, *J* = 8.2 Hz, 1H, H-2), 6.95 (d, *J* = 8.3 Hz, 1H, H-3). ^13^C NMR (101 MHz, CDCl_3_) δ (ppm) 157.0 (*C*O), 149.1 (C-8), 144.5 (C-5a), 141.9 (C-4a), 138.8 (C-7), 131.0 (C-2), 128.1 (C-1), 127.3 (C-6), 120.5 (C-3), 114.6 (C-4), 112.7 (C-10a), 105.3 (C-9), 56.4 (O*C*H_3_).

#### 3.2.6. Synthesis of 2-Chloro-*N*-(1-chloro-11-oxo-11*H*-pyrido[2,1-*b*]quinazolin-4-yl)acetamide (**8a**)

Chloroacetyl chloride (0.12 mL, 1.55 mmol) was added to a suspension of compound **7a** (320 mg, 1.04 mmol) and Na_2_CO_3_ (415 mg, 3.11 mmol) in a mixture of THF (15 mL) and DCM (2 mL) and the resulting suspension was stirred at room temperature for 15 min. After completion of the reaction, the mixture was evaporated to dryness and the crude residue was dissolved in DCM, washed with 5% Na_2_CO_3_ solution, dried (anh. Na_2_SO_4_), and vacuum evaporated to afford 360 mg of the title compound **9a**, which was practically pure and used for the next step without any further purification. Yield: 94%. Mp.: >230 °C (EtOAc). ^1^H NMR (600 MHz, CDCl_3_) δ (ppm) 10.58 (brs, 1H, D_2_O exch., N*H*CO), 8.91 (d, *J* = 7.0 Hz, 1H, H-9), 8.76 (d, *J* = 8.3 Hz, 1H, H-3), 7.61 (t, *J* = 6.7 Hz, 1H, H-7), 7.51 (d, *J* = 8.9 Hz, 1H, H-6), 7.44 (d, *J* = 8.4 Hz, 1H, H-2), 6.95 (t, *J* = 6.42 Hz, 1H, H-8), 4.29 (s, 2H, COC*H*_2_). ^13^C NMR (151 MHz, CDCl_3_) δ (ppm) 164.4 (NH*C*O), 156.6 (*C*O), 147.7 (C-5a), 140.8 (C-4a), 135.8 (C-7), 131.3 (C-4), 128.4 (C-1), 127.5 (C-2), 127.1 (C-9), 126.3 (C-6), 121.8 (C-3), 113.6 (C-8), 112.8 (C-10a), 43.5 (CO*C*H_2_).

#### 3.2.7. Synthesis of 2-Chloro-*N*-(1-chloro-8-methoxy-11-oxo-11*H*-pyrido[2,1-*b*]quinazolin-4-yl)acetamide (**8b**)

The title compound was synthesized by an analogous procedure as described for the preparation of **8a**, starting from **7b**. Yield: 94%. Mp.: >230 °C (EtOAc). ^1^H NMR (400 MHz, CDCl_3_) δ (ppm) 10.59 (brs, 1H, N*H*CO, D_2_O exch.), 8.74 (d, *J* = 8.5 Hz, 1H, H-3), 8.42 (d, *J* = 2.3 Hz, 1H, H-9), 7.52 (d, *J* = 9.7 Hz, H-7), 7.47–7.44 (m, 2H, H-2, H-6), 4.30 (s, 3H, COC*H*_2_), 3.93 (s, 3H, OC*H*_3_). ^13^C NMR (151 MHz, CDCl_3_) δ (ppm) 164.4 (NH*C*O), 156.3 (*C*O), 148.6 (C-8), 145.2 (C-5a), 140.5 (C-4a), 132.5 (C-7), 131.2 (C-4), 128.1 (C-2, C-6), 127.6 (C-1), 121.4 (C-3), 112.1 (C-10a), 105.7 (C-9), 56.5 (O*C*H_3_), 43.5 (CO*C*H_2_).

#### 3.2.8. Synthesis of 3-Chloro-*N*-(1-chloro-11-oxo-11*H*-pyrido[2,1-*b*]quinazolin-4-yl)propanamide (**9a**)

The title compound was synthesized by an analogous procedure as described for the preparation of **8a**, starting from **7a** in reaction with 3-chloropropionyl chloride. Yield: 82%. Mp.: >230 °C (EtOAc). ^1^H NMR (600 MHz, CDCl_3_) δ (ppm) 9.55 (brs, 1H, D_2_O exch., N*H*), 8.92 (d, *J* = 7.4 Hz, 1H, H-9), 8.82 (d, *J* = 8.5 Hz, 1H, H-3), 7.62 (t, *J* = 7.5 Hz, 1H, H-7), 7.53 (d, *J* = 9.1 Hz, 1H, H-6), 7.44 (d, *J* = 8.6 Hz, 1H, H-2), 6.96 (t, *J* = 6.3 Hz, 1H, H-8), 3.95 (t, *J* = 6.5 Hz, 2H, COCH_2_C*H*_2_), 3.02 (t, *J* = 6.5 Hz, 2H, COC*H*_2_CH_2_). ^13^C NMR (151 MHz, CDCl_3_) δ (ppm) 168.0 (NH*C*O), 156.6 (*C*O), 147.5 (C-5a), 140.1 (C-4a), 135.7 (C-7), 131.9 (C-4), 127.6 (C-1, C-2), 127.1 (C-9), 126.0 (C-6), 122.0 (C-3), 113.5 (C-8), 112.7 (C-10a), 41.1 (COCH_2_*C*H_2_), 39.8 (CO*C*H_2_CH_2_).

#### 3.2.9. Synthesis of 3-Chloro-*N*-(1-chloro-8-methoxy-11-oxo-11*H*-pyrido[2,1-*b*]quinazolin-4-yl)propanamide (**9b**)

The title compound was synthesized by an analogous procedure as described for the preparation of **9a**, starting from **7b**. Yield: 80%. Mp.: 154–156 °C (EtOAc). ^1^H NMR (600 MHz, DMSO-*d6*) δ (ppm) 11.07 (brs, 1H, D_2_O exch., N*H*CO), 8.61 (d, *J* = 8.5 Hz, 1H, H-3), 8.35 (d, *J* = 1.9 Hz, 1H, H-9), 7.50–7.45 (m, 2H, H-6, H-7), 7.39 (d, *J* = 8.5 Hz, 1H, H-2), 3.92 (s, 3H, OC*H*_3_), 3.81 (t, *J* = 4.9 Hz, 2H, COCH_2_C*H*_2_), 3.74 (t, *J* = 4.3 Hz, 2H, COC*H_2_*CH_2_). ^13^C NMR (151 MHz, DMSO-*d_6_*) δ (ppm) 168.2 (NH*C*O), 156.6 (*C*O), 149.7 (C-8), 145.3 (C-5a), 140.0 (C-4a), 132.6 (C-7), 132.1 (C-4), 127.9 (C-2), 127.5 (C-1), 127.0 (C-6), 121.6 (C-3), 112.3 (C-10a), 106.0 (C-9), 56.7 (O*C*H_3_), 41.3 (COCH*_2_C*H_2_), 40.0 (CO*C*H*_2_*CH_2_).

#### 3.2.10. Synthesis of *N*-(1-Chloro-11-oxo-11*H*-pyrido[2,1-*b*]quinazolin-4-yl)-2-(dimethylamino)acetamide (**1a**)

To a solution of compound **8a** (0.15 mmol) in EtOH absolute (20 mL), dimethylamine (0.27 mL, 1.5 mmol, 5.6 M ethanolic solution) was added and the resulting mixture was refluxed for 24 h. After completion of the reaction, the solvent was vacuum evaporated, and the residue was purified by flash chromatography (silica gel) using a mixture of DCM/MeOH as the eluent (100:0 to 94:6) to obtain the title product. Yield: 73%. Mp.: >230 °C (MeOH). ^1^H NMR (600 MHz, CDCl_3_) δ (ppm) 10.76 (brs, 1H, D_2_O exch., N*H*CO), 8.88 (d, *J* = 7.7 Hz, 1H, H-9), 8.77 (d, *J* = 8.5 Hz, 1H, H-3), 7.57 (t, *J* = 7.6 Hz, 1H, H-7), 7.52 (d, *J* = 9.0 Hz, 1H, H-6), 7.41 (d, *J* = 8.5 Hz, 1H, H-2), 6.92 (t, *J* = 6.3 Hz, 1H, H-8), 3.42 (s, 2H, COC*H*_2_), 2.61 (s, 3H, NH(C*H_3_*)_2_). ^13^C NMR (151 MHz, CDCl_3_) δ (ppm) 168.1 (NH*C*O), 156.7 (*C*O), 147.5 (C-5a), 140.9 (C-4a), 135.4 (C-7), 132.0 (C-4), 127.6 (C-2), 127.6 (C-1), 127.0 (C-9), 126.5 (C-6), 122.1 (C-3), 113.4 (C-8), 112.8 (C-10a), 63.2 (CO*C*H_2_), 45.7 (NH(*C*H_3_)_2_).

#### 3.2.11. Synthesis of *N*-(1-Chloro-8-methoxy-11-oxo-11*H*-pyrido[2,1-*b*]quinazolin-4-yl)-2-(dimethylamino)acetamide (**1b**)

The title compound was synthesized by an analogous procedure as described for the preparation of **1a**, starting from **8b**. Yield: 81%. Mp.: >230 °C (MeOH). ^1^H NMR (600 MHz, CDCl_3_) δ (ppm) 11.18 (brs, 1H, D_2_O exch., N*H*CO), 8.76 (d, *J* = 8.5 Hz, 1H, H-3), 8.40 (d, *J* = 2.6 Hz, 1H, H-9), 7.47 (dd, *J* = 9.7, 2.6 Hz, 1H, H-7), 7.48–7.40 (m, 2H, H-2, H-6), 3.92 (s, 3H, OC*H*_3_), 3.30 (s, 2H, COC*H*_2_), 2.54 (s, 3H, NH(C*H_3_*)_2_). ^13^C NMR (151 MHz, CDCl_3_) δ (ppm) 169.0 (NH*C*O), 156.5 (CO), 149.4 (C-8), 145.1 (C-5a), 140.4 (C-4α), 132.0 (C-7), 132.1 (C-4), 127.7 (C-2), 127.2 (C-6), 127.1 (C-1), 121.4 (C-3), 112.2 (C-10a), 105.6 (C-9), 63.8 (CO*C*H_2_), 56.4 (O*C*H_3_), 46.1 (NH(*C*H_3_)_2_).

#### 3.2.12. Synthesis of *N*-(1-Chloro-11-oxo-11*H*-pyrido[2,1-*b*]quinazolin-4-yl)-2-(diethylamino)acetamide (**1c**)

The title compound was synthesized by an analogous procedure as described for the preparation of **1a**, starting from **8a**. Yield: 45%. Mp.: 170–172 °C (MeOH). ^1^H NMR (600 MHz, CDCl_3_) δ (ppm) 11.30 (brs, 1H, D_2_O exch., N*H*CO), 8.91 (d, *J* = 7.4 Hz, 1H, H-9), 8.85 (d, *J* = 8.5 Hz, 1H, H-3), 7.58 (t, *J* = 7.6 Hz, 1H, H-7), 7.45 (m, 2H, H-2, H-6), 6.92 (t, *J* = 6.2 Hz, 1H, H-8), 3.28 (s, 2H, COC*H*_2_), 2.72 (q, *J* = 7.1 Hz, 4H, NH(C*H_2_*CH_3_)_2_), 1.19 (t, *J* = 7.1 Hz, 6H, NH(CH_2_C*H*_3_)_2_). ^13^C NMR (151 MHz, CDCl_3_) δ (ppm) 171.2 (NH*C*O), 156.9 (*C*O), 147.4 (C-5a), 140.9 (C-4a), 135.3 (C-7), 132.5 (C-4), 127.8 (C-2), 127.1 (C-1), 127.0 (C-9), 126.3 (C-6), 121.7 (C-3), 113.3 (C-8), 112.9 (C-10a), 59.4 (CO*C*H_2_), 48.8 (NH(*C*H_2_CH_3_)_2_), 13.0 (NH(CH_2_*C*H_3_)_2_).

#### 3.2.13. Synthesis of *N*-(1-Chloro-8-methoxy-11-oxo-11*H*-pyrido[2,1-*b*]quinazolin-4-yl)-2-(diethylamino)acetamide (**1d**)

The title compound was synthesized by an analogous procedure as described for the preparation of **1a**, starting from **8b**. Yield: 60%. Mp.: 207–209 °C (MeOH). ^1^H NMR (400 MHz, CDCl_3_) δ (ppm) 11.26 (brs, 1H, D_2_O exch., N*H*CO), 8.79 (d, *J* = 8.5 Hz, 1H, H-3), 8.39 (d, *J* = 1.9 Hz, 1H, H-9), 7.44–7.41 (m, 3H, H-2, H-6, H-7), 3.30 (s, 2H, COC*H*_2_), 2.73 (q, *J* = 5.6 Hz, 4H, NH(C*H_2_*CH_3_)_2_), 1.19 (t, *J* = 7.1 Hz, 6H, NH(CH_2_C*H*_3_)_2_). ^13^C NMR (101 MHz, CDCl_3_) δ (ppm) 164.3 (NH*C*O), 156.5 (*C*O), 149.3 (C-8), 145.1 (C-5a), 140.4 (C-4a), 132.4 (C-4), 132.0 (C-7), 127.8 (C-2), 127.5 (C-1), 127.0 (C-6), 121.1 (C-3), 112.2 (C-10a), 105.6 (C-9), 59.1 (CO*C*H_2_), 56.4 (O*C*H_3_), 48.8 (NH(*C*H_2_CH_3_)_2_), 12.9 (NH(CH_2_*C*H_3_)_2_).

#### 3.2.14. Synthesis of *N*-(1-Chloro-11-oxo-11*H*-pyrido[2,1-*b*]quinazolin-4-yl)-2-(4-methylpiperazin-1-yl)acetamide (**1e**)

The title compound was synthesized by an analogous procedure as described for the preparation of **1a**, starting from **8a**. Yield: 76%. Mp.: >230 °C (MeOH). ^1^H NMR (600 MHz, CDCl_3_) δ (ppm) 11.09 (brs, 1H, D_2_O exch., N*H*CO), 8.88 (d, *J* = 7.3 Hz, 1H, H-9), 8.79 (d, *J* = 8.5 Hz, 1H, H-3), 7.60 (t, *J* = 7.4 Hz, 1H, H-7), 7.50 (d, *J* = 9.0 Hz, 1H, H-6), 7.39 (d, *J* = 8.5 Hz, 1H, H-2), 6.39 (t, *J* = 7.0 Hz, 1H, H-8), 3.27 (s, 2H, COC*H*_2_), 2.75 (brs, 4H, C*H*_2_-3′, C*H*_2_-5′), 2.66 (brs, 4H, C*H*_2_-2′, C*H*_2_-6′), 2.41 (C*H_3_*). ^13^C NMR (151 MHz, CDCl_3_) δ (ppm) 169.2 (NH*C*O), 156.7 (*C*O), 147.4 (C-5a), 140.6 (C-4a), 135.4 (C-7), 132.2 (C-4), 127.7 (C-2), 127.2 (C-1), 127.1 (C-9), 125.9 (C-6), 121.6 (C-3), 113.3 (C-8), 112.8 (C-10a), 62.3 (CO*C*H_2_), 55.8 (*C*H_2_-2′, *C*H_2_-6′), 53.3 (*C*H_2_-3′, *C*H_2_-5′), 46.3 (*C*H_3_).

#### 3.2.15. Synthesis of *N*-(1-Chloro-8-methoxy-11-oxo-11*H*-pyrido[2,1-*b*]quinazolin-4-yl)-2-(4-methylpiperazin-1-yl)acetamide (**1f**)

The title compound was synthesized by an analogous procedure as described for the preparation of **1a**, starting from **8b**. Yield: 57%. Mp.: >230 °C (MeOH). ^1^H NMR (600 MHz, CDCl_3_) δ (ppm) 11.12 (brs, 1H, D_2_O exch., N*H*CO), 8.81 (d, *J* = 8.5 Hz, 1H, H-3), 8.42 (d, *J* = 2.0 Hz, 1H, H-9), 7.50 (dd, *J* = 9.7 Hz, 1H, H-7), 7.46–7.43 (m, 2H, H-2, H-6), 3.93 (s, 3H, OC*H*_3_), 3.27 (s, 2H, COC*H*_2_), 2.75 (brs, 4H, C*H*_2_-3′, C*H*_2_-5′), 2.65 (brs, 4H, C*H*_2_-2′, C*H*_2_-6′), 2.41 (C*H*_3_). ^13^C NMR (151 MHz, CDCl_3_) δ (ppm) 169.2 (NH*C*O), 156.5 (*C*O), 149.5 (C-8), 145.1 (C-5a), 140.3 (C-4a), 132.3 (C-4), 132.2 (C-7), 127.9 (C-2), 127.0 (C-1), 126.7 (C-6), 121.1 (C-3), 112.3 (C-10a), 105.7 (C-9), 62.4 (CO*C*H_2_), 56.5 (O*C*H_3_), 55.9 (*C*H_2_-2′, *C*H_2_-6′), 53.5 (*C*H_2_-3′, *C*H_2_-5′), 46.5 (*C*H_3_).

#### 3.2.16. Synthesis of *N*-(1-Chloro-11-oxo-11*H*-pyrido[2,1-*b*]quinazolin-4-yl)-2-(4-(2-methoxyethyl)piperazin-1-yl)acetamide (**1g**)

The title compound was synthesized by an analogous procedure as described for the preparation of **1a**, starting from **8a**. Yield: 94%. Mp.: 216–218 °C (MeOH). ^1^H NMR (600 MHz, CDCl_3_) δ (ppm) 11.05 (brs, 1H, D_2_O exch., N*H*CO), 8.81 (d, *J* = 7.3 Hz, 1H, H-9), 8.72 (d, *J* = 8.5 Hz, 1H, H-3), 7.55 (t, *J* = 7.7 Hz, 1H, H-7), 7.43 (d, *J* = 9.0 Hz, 1H, H-6), 7.32 (d, *J* = 8.5 Hz, 1H, H-2), 6.89 (t, *J* = 6.7 Hz, 1H, H-8), 3.54 (t, *J* = 5.6 Hz, 2H, CH_2_C*H*_2_O), 3.35 (s, 3H, OC*H_3_*), 3.22 (s, 2H, COC*H*_2_), 2.75 (brs, 8H, C*H*_2_-2′, C*H*_2_-3′, C*H*_2_-5′, C*H*_2_-6′), 2.66 (t, *J* = 5.6 Hz 2H, C*H_2_*CH_2_O). ^13^C NMR (151 MHz, CDCl_3_) δ (ppm) 169.2 (NH*C*O), 156.5 (*C*O), 147.2 (C-5a), 140.5 (C-4a), 135.4 (C-7), 132.1 (C-4), 127.5 (C-2), 126.9 (C-1), 126.9 (C-9), 125.9 (C-6), 121.4 (C-3), 113.3 (C-8), 112.6 (C-10a), 70.4 (CH_2_*C*H_2_O), 62.3 (CO*C*H_2_), 59.0 (O*C*H_3_), 58.1 (*C*H_2_CH_2_O), 54.3 (*C*H_2_-2′, *C*H_2_-6′), 53.3 (*C*H_2_-3′, *C*H_2_-5′).

#### 3.2.17. Synthesis of *N*-(1-Chloro-8-methoxy-11-oxo-11*H*-pyrido[2,1-*b*]quinazolin-4-yl)-2-(4-(2-methoxyethyl)piperazin-1-yl)acetamide (**1h**)

The title compound was synthesized by an analogous procedure as described for the preparation of **1a**, starting from **8b**. Yield: 89%. Mp.: >230 °C (MeOH). ^1^H NMR (600 MHz, CDCl_3_) δ (ppm) 11.00 (brs, 1H, D_2_O exch., N*H*CO), 8.75 (d, *J* = 8.5 Hz, 1H, H-3), 8.37 (d, *J* = 2.0 Hz, 1H, H-9), 7.46–7.41 (m, 2H, H-6, H-7), 7.39 (d, *J* = 8.5 Hz, 1H, H-2), 3.92 (s, 3H, OC*H_3_*), 3.66 (brs, 2H, CH_2_C*H*_2_O), 3.38 (s, 3H, C*H_3_*), 3.29 (s, 2H, COC*H*_2_), 2.85 (brs, 10H, C*H*_2_-2′, C*H*_2_-3′, C*H*_2_-5′, C*H*_2_-6′, C*H_2_*CH_2_O). ^13^C NMR (151 MHz, CDCl_3_) δ (ppm) 168.9 (NH*C*O), 156.4 (*C*O), 149.3 (C-8), 145.0 (C-5a), 140.2 (C-4a), 132.2 (C-7), 132.1 (C-4), 127.7 (C-2), 127.0 (C-1), 126.7 (C-6), 121.1 (C-3), 112.1 (C-10a), 105.7 (C-9), 69.7 (CH_2_*C*H_2_O), 62.2 (CO*C*H_2_), 59.1 (*C*H_3_), 57.8 (*C*H_2_CH_2_O), 54.0 (*C*H_2_-2′, *C*H_2_-6′), 52.6 (*C*H_2_-3′, *C*H_2_-5′).

#### 3.2.18. Synthesis of *N*-(1-Chloro-11-oxo-11*H*-pyrido[2,1-*b*]quinazolin-4-yl)-2-((2-(2-hydroxyethoxy)ethyl)amino)acetamide (**1i**)

The title compound was synthesized by an analogous procedure as described for the preparation of **1a**, starting from **8a**. Yield: 71%. Mp.: 181–183 °C (MeOH). ^1^H NMR (600 MHz, CDCl_3_) δ (ppm) 10.94 (brs, 1H, D_2_O exch., N*H*CO), 8.89 (d, *J* = 7.3 Hz, 1H, H-9), 8.5 (d, *J* = 7.4 Hz, 1H, H-3), 7.59 (t, *J* = 6.5 Hz, 1H, H-7), 7.48 (d, *J* = 9.0 Hz, 1H, H-6), 7.41 (d, *J* = 8.5 Hz, 1H, H-2), 6.93 (t, *J* = 6.21 Hz, 1H, H-8), 3.77–3.73 (m, 4H, NHCH_2_C*H_2_*, CH_2_C*H*_2_OH), 3.61 (t, *J* = 4.7 Hz, 2H, C*H*_2_CH_2_OH), 3.60 (s, 2H, COC*H*_2_), 3.00 (t, *J* = 5.0 Hz, 2H, NHC*H*_2_CH_2_). ^13^C NMR (151 MHz, CDCl_3_) δ (ppm) 170.6 (NH*C*O), 156.7 (*C*O), 147.5 (C-5a), 140.7 (C-4a), 135.5 (C-7), 132.1 (C-4), 127.7 (C-2), 127.4 (C-1), 127.1 (C-9), 126.2 (C-6), 121.9 (C-3), 113.4 (C-8), 112.8 (C-10a), 72.6 (*C*H_2_CH_2_OH), 71.0 (NHCH_2_*C*H_2_), 62.0 (CH_2_*C*H_2_OH), 53.7 (CO*C*H_2_), 49.5 (NH*C*H_2_CH_2_).

#### 3.2.19. Synthesis of *N*-(1-Chloro-8-methoxy-11-oxo-11*H*-pyrido[2,1-*b*]quinazolin-4-yl)-2-((2-(2-hydroxyethoxy)ethyl)amino)acetamide (**1j**)

The title compound was synthesized by an analogous procedure as described for the preparation of **1a**, starting from **8b**. Yield: 36%. Mp.: 176–178 °C (MeOH). ^1^H NMR (600 MHz, DMSO-*d_6_*) δ (ppm) 11.18 (brs, 1H, D_2_O exch., N*H*CO), 8.71 (d, *J* = 8.5 Hz, 1H, H-3), 8.33 (d, *J* = 2.0 Hz, 1H, H-9), 7.74 (dd, *J* = 9.7, 2.6 Hz, 1H, H-7), 7.51 (d, *J* = 9.7 Hz, 1H, H-6), 7.49 (d, *J* = 8.5 Hz, 1H, H-2), 3.92 (s, 3H, OC*H*_3_), 3.64 (t, 2H, *J* = 5.5 Hz, 2H, NHCH_2_C*H*_2_), 3.50 (t, *J* = 5.2 Hz, 2H, CH_2_C*H*_2_OH), 3.46–3.43 (m, 4H, COC*H*_2_, C*H*_2_CH_2_OH), 2.82 (t, *J* = 5.0 Hz, 2H, NHC*H*_2_CH_2_). ^13^C NMR (151 MHz, DMSO-*d*_6_) δ (ppm) 171.1 (NH*C*O), 155.5 (*C*O), 148.9 (C-8), 145.0 (C-5a), 139.8 (C-4a), 132.5 (C-7), 132.1 (C-4), 126.7 (C-2, C-6), 124.9 (C-1), 119.7 (C-3), 111.4 (C-10a), 105.7 (C-9), 72.7 (*C*H_2_CH_2_OH), 70.2 (NHCH_2_*C*H_2_), 62.2 (CH_2_*C*H_2_OH), 56.3 (O*C*H_3_), 53.0 (CO*C*H_2_), 49.9 (NH*C*H_2_CH_2_).

#### 3.2.20. Synthesis of *N*-(1-Chloro-11-oxo-11*H*-pyrido[2,1-*b*]quinazolin-4-yl)-3-(dimethylamino)propanamide (**2a**)

The title compound was synthesized by an analogous procedure as described for the preparation of **1a**, starting from **9a**. Yield: 56%. Mp.: 162–164 °C (MeOH). ^1^H NMR (600 MHz, CDCl_3_) δ (ppm) 11.29 (brs, 1H, D_2_O exch., N*H*CO), 8.89 (d, *J* = 7.7 Hz, 1H, H-9), 8.85 (d, *J* = 8.6 Hz, 1H, H-3), 7.52 (t, *J* = 7.6 Hz, 1H, H-7), 7.45 (d, *J* = 9.1 Hz, 1H, H-6), 7.42 (d, *J* = 8.6 Hz, 1H, H-2), 6.91 (t, *J* = 6.3 Hz, 1H, H-8), 2.81 (t, *J* = 5.2 Hz, 2H, COCH_2_C*H*_2_), 2.72 (t, *J* = 5.8 Hz, 2H, COC*H*_2_CH_2_), 2.49 (s, 3H, NH(C*H*_3_)_2_). ^13^C NMR (151 MHz, CDCl_3_) δ (ppm) 171.0 (NH*C*O), 157.0 (*C*O), 147.2 (C-5a), 140.8 (C-4a), 135.3 (C-7), 133.4 (C-4), 127.8 (C-2), 127.1 (C-9), 127.0 (C-1), 126.1 (C-6), 122.6 (C-3), 113.3 (C-8), 112.8 (C-10a), 54.2 (COCH_2_*C*H_2_), 45.1 (NH(*C*H_3_)_2_), 35.0 (CO*C*H_2_CH_2_).

#### 3.2.21. Synthesis of *N*-(1-Chloro-8-methoxy-11-oxo-11*H*-pyrido[2,1-*b*]quinazolin-4-yl)-3-(dimethylamino)propanamide (**2b**)

The title compound was synthesized by an analogous procedure as described for the preparation of **1a**, starting from **9b**. Yield: 81%. Mp.: 203–205 °C (MeOH). ^1^H NMR (600 MHz, CDCl_3_) δ (ppm) 11.18 (brs, 1H, D_2_O exch., N*H*CO), 8.80 (d, *J* = 8.6 Hz, 1H, H-3), 8.38 (d, *J* = 2.0 Hz, 1H, H-9), 7.41–7.40 (m, 3H, H-2, H-6, H-7), 3.91 (s, 3H, OC*H*_3_), 2.84 (t, 2H, *J* = 6.0 Hz, COC*H*_2_CH_2_), 2.73 (t, 2H, *J* = 6.2 Hz, COC*H*_2_CH_2_), 2.50 (s, 3H, NH(C*H*_3_)_2_). ^13^C NMR (151 MHz, CDCl_3_) δ (ppm) 170.8 (NH*C*O), 156.5 (*C*O), 149.3 (C-8), 144.8 (C-5a), 140.3 (C-4a), 133.2 (C-4), 131.9 (C-7), 127.8 (C-2), 127.0 (C-1), 126.8 (C-6), 121.9 (C-3), 112.1 (C-10a), 105.6 (C-9), 56.4 (OC*H*_3_), 54.9 (COCH_2_*C*H_2_), 45.1 (NH(*C*H_3_)_2_), 34.9 (COCH_2_*C*H_2_).

#### 3.2.22. Synthesis of *N*-(1-Chloro-11-oxo-11*H*-pyrido[2,1-*b*]quinazolin-4-yl)-3-(diethylamino)propanamide (**2c**)

The title compound was synthesized by an analogous procedure as described for the preparation of **1a**, starting from **9a**. Yield: 90%. Mp.: 162–164 °C (MeOH). ^1^H NMR (600 MHz, CDCl_3_) δ (ppm) 11.23 (brs, 1H, D_2_O exch., N*H*CO), 8.89–8.88 (m, 2H, H-3, H-9), 7.56 (t, *J*= 7.6 Hz, 1H, H-7), 7.44 (d, *J* = 9.0 Hz, 1H, H-6), 7.41 (d, *J*= 8.6 Hz, 1H, H-2), 6.90 (t, *J* = 6.3 Hz, 1H, H-8), 2.89 (t, *J* = 6.1 Hz, 2H, COCH_2_C*H*_2_), 2.77 (q, *J* = 7.1 Hz, 4H, NH(C*H_2_*CH_3_)_2_), 2.69 (t, *J* = 6.2 Hz, 2H, COC*H*_2_CH_2_), 1.16 (t, *J* = 7.2 Hz, 6H, NH(CH_2_C*H*_3_)_2_). ^13^C NMR (151 MHz, CDCl_3_) δ (ppm) 171.6 (NH*C*O), 156.9 (*C*O), 147.1 (C-5a), 140.7 (C-4a), 135.2 (C-7), 133.5 (C-4), 127.8 (C-2), 126.9 (C-1), 127.1 (C-9), 125.9 (C-6), 122.7 (C-3), 113.2 (C-8), 112.8 (C-10a), 48.4 (COCH_2_*C*H_2_), 46.8 (NH(*C*H_2_CH_3_)_2_), 35.0 (CO*C*H_2_CH_2_), 11.3 (NH(CH_2_*C*H_3_)_2_).

#### 3.2.23. Synthesis of *N*-(1-Chloro-8-methoxy-11-oxo-11*H*-pyrido[2,1-*b*]quinazolin-4-yl)-3-(diethylamino)propanamide (**2d**)

The title compound was synthesized by an analogous procedure as described for the preparation of **1a**, starting from **9b**. Yield: 60%. Mp.: >230 °C (MeOH). ^1^H NMR (600 MHz, CDCl_3_) δ (ppm) 11.30 (brs, 1H, D_2_O exch., N*H*CO), 8.86 (d, *J* = 8.6 Hz, 1H, H-3), 8.39 (d, *J* = 2.0 Hz, 1H, H-9), 7.43–7.41 (m, 3H, H-2, H-6, H-7), 3.91 (s, 3H, OC*H_3_*), 2.87 (t, 2H, *J* = 5.9 Hz, COCH_2_C*H*_2_), 2.75 (q, *J* = 7.1 Hz, 4H, NH(C*H_2_*CH_3_)_2_), 2.68 (t, 2H, *J* = 6.0 Hz, COC*H*_2_CH_2_), 1.15 (t, *J* = 7.1 Hz, 6H, NH(CH_2_C*H_3_*)_2_).^13^C NMR (151 MHz, CDCl_3_) δ (ppm) 171.3 (NH*C*O), 156.6 (*C*O), 149.3 (C-8), 144.7 (C-5a), 140.3 (C-4a), 133.3 (C-4), 132.0 (C-7), 127.8 (C-2), 126.7 (C-1), 126.6 (C-6), 122.0 (C-3), 112.2 (C-10a), 105.6 (C-9), 56.4 (O*C*H_3_), 48.4 (COCH_2_*C*H_2_), 46.7 (NH(*C*H_2_CH_3_)_2_), 34.9 (CO*C*H_2_CH_2_), 11.1 (NH(CH_2_*C*H_3_)_2_).

#### 3.2.24. Synthesis of *N*-(1-Chloro-11-oxo-11*H*-pyrido[2,1-*b*]quinazolin-4-yl)-3-(4-methylpiperazin-1-yl)propanamide (**2e**)

The title compound was synthesized by an analogous procedure as described for the preparation of **1a**, starting from **9a**. Yield: 70%. Mp.: 195–197 °C (MeOH). ^1^H NMR (600 MHz, CDCl_3_) δ (ppm) 10.12 (brs, 1H, D_2_O exch., N*H*CO), 8.88 (d, *J* = 7.3 Hz, 1H, H-9), 8.76 (d, *J* = 8.6 Hz, 1H, H-3), 7.57 (t, *J* = 7.6 Hz, 1H, H-7), 7.46 (d, *J* = 9.0 Hz, 1H, H-6), 7.40 (d, *J* = 8.6 Hz, 1H, H-2), 6.92 (t, *J* = 6.2 Hz, 1H, H-8), 2.88 (t, *J* = 6.6 Hz, 2H, COCH_2_C*H*_2_), 2.72 (m, 6H, COC*H*_2_CH_2_, C*H*_2_-3′, C*H*_2_-5′), 2.57 (brs, 4H, C*H*_2_-2′, C*H*_2_-6′), 2.32 (s, 3H, C*H_3_*). ^13^C NMR (151 MHz, CDCl_3_) δ (ppm) 170.7 (NH*C*O), 156.7 (*C*O), 147.3 (C-5a), 140.6 (C-4a), 135.4 (C-7), 132.2 (C-4), 127.7 (C-2), 127.3 (C-1), 127.1 (C-9), 125.9 (C-6), 122.7 (C-3), 113.3 (C-8), 112.8 (C-10a), 54.9 (*C*H_2_-2′, *C*H_2_-6′), 53.8 (COCH_2_*C*H_2_), 52.8 (*C*H_2_-3′, *C*H_2_-5′), 45.9 (*C*H_3_), 35.1 (CO*C*H_2_CH_2_).

#### 3.2.25. Synthesis of *N*-(1-Chloro-8-methoxy-11-oxo-11*H*-pyrido[2,1-*b*]quinazolin-4-yl)-3-(4-methylpiperazin-1-yl)propanamide (**2f**)

The title compound was synthesized by an analogous procedure as described for the preparation of **1a**, starting from **9b**. Yield: 86%. Mp.: 200–202 °C (MeOH). ^1^H NMR (600 MHz, CDCl_3_) δ (ppm) 10.08 (brs, 1H, D_2_O exch., N*H*CO), 8.72 (d, *J* = 8.5 Hz, 1H, H-3), 8.36 (d, *J* = 2.2 Hz, 1H, H-9), 7.42–7.37 (m, 3H, H-2, H-6, H-7), 3.90 (s, 3H, OC*H*_3_), 2.87 (t, *J* = 6.6 Hz, 2H, COCH_2_C*H*_2_), 2.71 (m, 6H, COC*H*_2_CH_2_, C*H*_2_-3′, C*H*_2_-5′), 2.57 (brs, 4H, C*H*_2_-2′, C*H*_2_-6′), 2.32 (C*H*_3_). ^13^C NMR (151 MHz, CDCl_3_) δ (ppm) 170.6 (NH*C*O), 156.4 (*C*O), 149.3 (C-8), 144.8 (C-5a), 140.1 (C-4a), 132.5 (C-4), 132.0 (C-7), 127.7 (C-2), 126.9 (C-1), 126.7 (C-6), 122.0 (C-3), 112.1 (C-10a), 105.6 (C-9), 56.4 (OC*H*_3_), 54.8 (*C*H_2_-2′, *C*H_2_-6′), 53.8 (COCH_2_*C*H_2_), 52.7 (*C*H_2_-3′, *C*H_2_-5′), 45.9 (*C*H_3_), 35.1 (CO*C*H_2_CH_2_).

#### 3.2.26. Synthesis of *N*-(1-Chloro-11-oxo-11*H*-pyrido[2,1-*b*]quinazolin-4-yl)-3-(4-(2-methoxyethyl)piperazin-1-yl)propanamide (**2g**)

The title compound was synthesized by an analogous procedure as described for the preparation of **1a**, starting from **9a**. Yield: 83%. Mp.: 177–179 °C (MeOH). ^1^H NMR (600 MHz, CDCl_3_) δ (ppm) 10.11 (brs, 1H, D_2_O exch., N*H*CO), 8.88 (d, *J* = 7.0 Hz, 1H, H-9), 8.76 (d, *J* = 8.6 Hz, 1H, H-3), 7.57 (t, *J* = 7.6 Hz, 1H, H-7), 7.47 (d, *J* = 9.1 Hz, 1H, H-6), 7.43 (d, *J* = 8.6 Hz, 1H, H-2), 6.92 (t, *J* = 6.3 Hz, 1H, H-8), 3.51 (t, *J* = 5.6 Hz, 2H, CH_2_C*H*_2_O), 3.32 (s, 3H, C*H_3_*), 2.88 (t, *J* = 6.7 Hz, 2H, COCH_2_C*H_2_*), 2.73 (m, 6H, COC*H*_2_CH_2_, C*H*_2_-3′, C*H*_2_-5′), 2.64 (brs, 4H, C*H*_2_-2′, C*H*_2_-6′), 2.59 (t, *J* = 5.6 Hz, 2H, C*H*_2_CH_2_O). ^13^C NMR (151 MHz, CDCl_3_) δ (ppm) 170.6 (NH*C*O), 156.7 (*C*O), 147.3 (C-5a), 140.6 (C-4a), 135.4 (C-7), 132.8 (C-4), 127.7 (C-2), 127.3 (C-1), 127.1 (C-9), 126.0 (C-6), 122.7 (C-3), 113.3 (C-8), 112.8 (C-10a), 70.2 (CH_2_*C*H_2_O), 59.0 (*C*H_3_), 57.9 (*C*H_2_CH_2_O), 53.9 (COCH_2_*C*H_2_), 53.3 (*C*H_2_-2′, *C*H_2_-6′), 52.8 (*C*H_2_-3′, *C*H_2_-5′), 35.1 (CO*C*H_2_CH_2_).

#### 3.2.27. Synthesis of *N*-(1-Chloro-8-methoxy-11-oxo-11*H*-pyrido[2,1-*b*]quinazolin-4-yl)-3-(4-(2-methoxyethyl)piperazin-1-yl)propanamide (**2h**)

The title compound was synthesized by an analogous procedure as described for the preparation of **1a**, starting from **9b**. Yield: 58%. Mp.: 174–176 °C (MeOH). ^1^H NMR (600 MHz, CD_3_OD) δ (ppm) 8.62 (d, *J* = 8.5 Hz, 1H, H-3), 8.36 (d, *J* = 2.3 Hz, 1H, H-9), 3.99 (s, 3H, OC*H*_3_), 3.55 (t, *J* = 5.5 Hz, 2H, CH_2_C*H_2_*O), 3.34 (s, 3H, C*H*_3_), 2.88 (t, *J* = 6.6 Hz, 2H, COCH_2_C*H_2_*), 2.79 (t, *J* = 6.6 Hz, 2H, COC*H*_2_CH_2,_), 2.68 (brs, 4H, C*H*_2_-3′, C*H*_2_-5′), 2.61 (m, 6H, C*H_2_*CH_2_O, C*H*_2_-2′, C*H*_2_-6′). ^13^C NMR (151 MHz, CD_3_OD) δ (ppm) 172.8 (NH*C*O), 157.3 (*C*O), 150.9 (C-8), 146.3 (C-5a), 141.5 (C-4a), 133.4 (C-4), 133.3 (C-7), 128.0 (C-2, C-6), 127.9 (C-1), 122.8 (C-3), 112.8 (C-10a), 106.5 (C-9), 70.9 (CH_2_*C*H_2_O), 58.5 (*C*H_2_CH_2_O), 56.9 (O*C*H_3_), 54.7 (COCH_2_*C*H_2_), 54.2 (*C*H_2_-2′, *C*H_2_-6′), 53.4 (*C*H_2_-3′, *C*H_2_-5′), 35.3 (CO*C*H_2_CH_2_).

#### 3.2.28. Synthesis of *N*-(1-Chloro-11-oxo-11*H*-pyrido[2,1-*b*]quinazolin-4-yl)-3-((2-(2-hydroxyethoxy)ethyl)amino)propanamide (**2i**)

The title compound was synthesized by an analogous procedure as described for the preparation of **1a**, starting from **9a**. Yield: 50%. Mp.: >230 °C (MeOH). ^1^H NMR (400 MHz, DMSO-*d*_6_) δ (ppm) 10.39 (brs, 1H, D_2_O exch., N*H*CO), 8.82 (dd, *J* = 7.4, 1.5 Hz, 1H, H-9), 8.65 (d, *J* = 8.6 Hz, 1H, H-3), 7.85 (t, *J* = 8.3 Hz, 1H, H-7), 7.61 (d, *J* = 9.0 Hz, 1H, H-6), 7.47 (d, *J* = 8.6 Hz, 1H, H-2), 7.16 (t, *J* = 6.3 Hz, 1H, H-8), 3.67 (t, *J* = 5.2 Hz, 2H, NHCH_2_C*H_2_*O), 3.52 (t, *J* = 4.3 Hz, 2H, CH*_2_*C*H*_2_OH), 3.48 (t, *J* = 4.7 Hz, 2H, C*H_2_*CH_2_OH), 3.20 (t, *J* = 6.8 Hz, 2H, COCH*_2_*C*H*_2_), 3.08 (t, *J* = 5.3 Hz, 2H, NHC*H_2_*CH_2_O), 2.96 (t, *J* = 6.9 Hz, 2H, COC*H*_2_). ^13^C NMR (151 MHz, DMSO-*d_6_*) δ (ppm) 169.6 (NH*C*O), 156.1 (*C*O), 147.2 (C-5a), 140.5 (C-4a), 132.5 (C-7), 126.9 (C-2), 126.7 (C-1), 126.5 (C-9), 125.6 (C-6), 121.9 (C-3), 114.2 (C-8), 112.2 (C-10a), 72.3 (*C*H_2_CH_2_OH), 67.4 (NHCH_2_*C*H_2_O), 60.1 (CH_2_*C*H_2_OH), 47.0 (NH*C*H_2_CH_2_O), 43.4 (COCH_2_*C*H_2_), 33.4 (CO*C*H_2_CH_2_).

#### 3.2.29. Synthesis of *N*-(1-Chloro-8-methoxy-11-oxo-11*H*-pyrido[2,1-*b*]quinazolin-4-yl)-3-((2-(2-hydroxyethoxy)ethyl)amino)propanamide (**2j**)

The title compound was synthesized by an analogous procedure as described for the preparation of **1a**, starting from **9b**. Yield: 36%. Mp.: 226–228 °C (MeOH). ^1^H NMR (600 MHz, CD_3_OD) δ (ppm) 8.61 (d, *J* = 8.5 Hz, 1H, H-3), 8.35 (d, *J* = 1.9 Hz, 1H, H-9), 7.50–7.45 (m, 2H, H-6, H-7), 7.39 (d, *J* = 8.5 Hz, 1H, H-2), 3.97 (s, 3H, OC*H*_3_), 3.81 (t, *J* = 4.9 Hz, 2H, NHCH_2_C*H*_2_O), 3.74 (t, *J* = 4.3 Hz, 2H, CH_2_C*H*_2_OH), 3.64 (t, *J* = 4.7 Hz, 2H, C*H*_2_CH_2_OH), 3.49 (t, *J* = 6.3 Hz, 2H, COCH_2_C*H*_2_), 3.35 (t, *J* = 5.0 Hz, 2H, NHC*H*_2_CH_2_O), 3.12 (t, *J* = 6.3 Hz, 2H, COC*H*_2_CH_2_). ^13^C NMR (151 MHz, CD_3_OD) δ (ppm) 170.5 (NH*C*O), 154.5 (*C*O), 151.1 (C-8), 146.7 (C-5a), 141.6 (C-4a), 133.1 (C-4), 133.6 (C-7), 133.1 (C-3), 128.4 (C-1), 127.9 (C-2, C-6), 122.9 (C-3), 112.9 (C-10a), 106.6 (C-9), 73.5 (*C*H_2_CH_2_OH), 66.6 (NHCH_2_*C*H_2_O), 62.0 (CH_2_*C*H_2_OH), 56.9 (O*C*H_3_), 49.6 (NH*C*H_2_CH_2_O), 44.7 (COCH_2_*C*H_2_), 33.3 (CO*C*H_2_CH_2_).

### 3.3. NMR Experiments

The quadruplex NMR sample was prepared at a concentration of 0.4 mM (in 0.6 mL (H_2_O/D_2_O 9:1) buffer solution with 70 mM KCl (Merck KGaA, Darmstadt, Germany), 25 mM potassium phosphate buffer for Pu22-T14T23 (pH = 6.9). The oligonucleotide was heated to 85 °C for 1 min and then cooled at room temperature overnight.

NMR spectra were recorded with Bruker AV 600 MHz spectrometer. ^1^H chemical shifts were referenced relative to external sodium 2,2-dimethyl-2-silapentane-5-sulfonate (DSS). Monodimensional proton spectra were recorded using the pulse-field gradient for H_2_O suppression. Stock solution of **2i** was prepared in DMSO-*d_6_*. ^1^H NMR titrations were performed at 25 °C by adding increasing amounts of ligand to the DNA at different ratios R = [drug]/[DNA]. The protons in the complex were assigned using NOESY and TOCSY experiments. Phase-sensitive NOESY spectra were acquired at 25 °C, in TPPI mode, with 2048 × 1024 complex FIDs. Mixing times ranged from 200 ms to 350 ms. TOCSY spectra were acquired with the use of a MLEV-17 spin-lock pulse (60 ms total duration). All spectra were transformed and weighed with a 90° shifted sine-bell squared function to 4 K × 4 K real data points. NOESY and TOCSY spectra were analyzed for the solution with R = [drug]/[DNA] = 4.0.

Pseudo two-dimensional DOSY experiments were acquired using the pulse program “stebpgp1s”; diffusion delay: 0.12–0.45 s; gradient pulse: 1.5 ms; number of increments: 64. Raw data were processed using the standard DOSY software present in the Bruker library (TOPSPIN v.1.3). A calibration curve was obtained using, as standards, samples with a range of MW from 180 to 23,500.

### 3.4. Modeling Studies

Molecular modeling studies were conducted using the NMR ensemble deposited in the Protein Data Bank for c-MYC, PDB accession code: 2L7V, https://doi.org/10.2210/pdb2L7V/pdb (accessed on 9 November 2011) [20]. The three-dimensional structure of the ligand was obtained by optimization with the Tinker molecular modeling software v.8.10 [22].

Molecular docking calculations for **2i** were performed by AutoDock 4.2 [23,24], using the LGA (Lamarckian genetic algorithm) together with a grid-based energy evaluation method to calculate grid maps, using a 80 Å × 80 Å × 80 Å box with a spacing of 0.01 Å. The phosphorus atoms in the DNA were parameterized using the Cornell parameters. The AutoDock Toolkit (ADT) [25] was used to add the Gasteiger–Marsili charges [26] to the ligands and the Addsol utility of AutoDock was used to set up the solvation parameters. The initial population for each molecule consisted of 150 randomly placed individuals, with a maximum number of 250 energy evaluations and an elitism value of 1, a mutation rate of 0.02, and a crossover rate of 0.80. The local search was conducted by applying the so-called pseudo-Solis and Wets algorithm with a maximum of 500 iterations per local search and 500 independent docking runs. The docking results were scored using an in-house version of the simpler intermolecular energy function based on the Weiner force field, and the lowest energy conformations at 5′- and 3′-end (differing by less than 1.0 Å in positional root-mean-square deviation (rmsd)) were collected.

The resulting complexes were placed at the center of a box (boundaries at 2.0 nm apart from all atoms) and solvated with TIP3P water molecules [27]. Amber ff99 force field [28] with bsc1 corrections [29] were used to describe the c-MYC G-quadruplex. To remove bad contacts, 1000 minimization steps were performed on the initial systems, followed by a heating ramp of short (100 ps) consecutive simulations. The production simulations consisted of 5 ns of Langevin molecular dynamics (LMD) [30,31] NPT equilibration at 298 K and 1 atm, as implemented in NAMD [32]. During this step, all bonds to hydrogen atoms were constrained using the SHAKE algorithm [33]. Water molecules were kept rigid with SETTLE [34], allowing for an integration time step of 0.002 ps. The electrostatic interactions were calculated using the particle mesh Ewald (PME) method (Coulomb cut-off radius of 1.2 nm) [35,36]. A Berendsen thermostat (coupling time of 0.1 ps) was applied to the systems [37]. Molecular graphics and analyses were performed with UCSF ChimeraX 1.16, developed by the Resource for Biocomputing, Visualization, and Informatics at the University of California, San Francisco, with support from National Institutes of Health R01-GM129325 and the Office of Cyber Infrastructure and Computational Biology, National Institute of Allergy and Infectious Diseases [38,39].

### 3.5. Antiproliferative Activity Evaluation

The antiproliferative activity of the compounds was evaluated using the human osteosarcoma U2OS cell line (ATCC HTB-96) [40,41], cultured in Mc Coy’s 5A medium plus 10% foetal bovine serum at 37 °C and 5% CO_2_. This cell line is telomerase (hTERT) negative and maintains telomere length via alternative lengthening of telomeres (ALT) pathway. Cytotoxic potency was assessed by a growth inhibition assay (CellTiter 96^®^ AQueous One Solution Cell Proliferation Assay MTS, Promega, Madison, WI, USA). Cells were seeded in a 96-well plate (2000 cells/well) and 24 h later exposed to the compounds (concentration range 1–100 µM). After 72 h of exposure, 3-(4,5-dimethylthiazol-2-yl)-5-(3-carboxymethoxyphenyl)-2-(4-sulfophenyl)-2H-tetrazolium salt was added to each well. The absorbance was measured using a FLUOstar OPTIMA plate reader (BMG Labtech GmbH, Offenburg, Germany) at 492 nm after 4 h of incubation at 37 °C in 5% CO_2_. The IC_50_ was defined as the drug concentration causing 50% cell growth inhibition, as determined by the dose-response curves.

RHPS4 and BMH21 were used as references. Besides binding to telomeric G4, RHPS4 targets also G4 structures positioned in promoters or introns of a series of genes implicated in cancer progression (e.g., MYC, VEGFR2) and stemness (e.g., CD133, CD44) [42]. Experiments were performed in triplicate.

## 4. Conclusions

Twenty novel compounds featuring the 11*H*-pyrido[2,1-*b*]quinazolin-11-one scaffold were synthesized and evaluated as potential c-MYC G4 ligands. All the new compounds exhibited moderate to good antiproliferative activity. Notably, compounds **2i** and **2j**, bearing a 2-((2-(2-hydroxyethoxy)ethyl)amino)acetyl group connected by an amido linkage to the quinazolinone skeleton, demonstrated the most promising antiproliferative activity in the low-micromolar range. Of particular interest, compound **2i**, displaying intriguing cytotoxic properties, underwent further investigation through NMR and docking experiments to assess its potential to interact with G-quadruplexes in the c-MYC promoter region. The findings suggest that compound **2i** binds to the Pu22 quadruplex, forming a 2:1 complex and positioning each molecule over the tetrads at the 3′- and 5′-ends. It can be asserted that the flat aromatic ring system inherent in 11*H*-pyrido[2,1-*b*]quinazolin-11-ones appears to be favorable for quadruplex recognition. The facile and high-yielding approach to constructing the tricyclic ring system could provide access to a diverse group of analogues with varying substitutions, thereby offering opportunities for further structural optimization. Furthermore, the molecular model developed for the complexes with DNA G4 structures holds potential as valuable inspiration for designing novel molecules that interact with c-MYC.

## Figures and Tables

**Table 1:** Structures of the synthesized compounds and their corresponding IC_50_ (μM) on U2OS cells.

Compounds	R_1_	R_2_	IC_50_ (μΜ) (±) SD ^a^
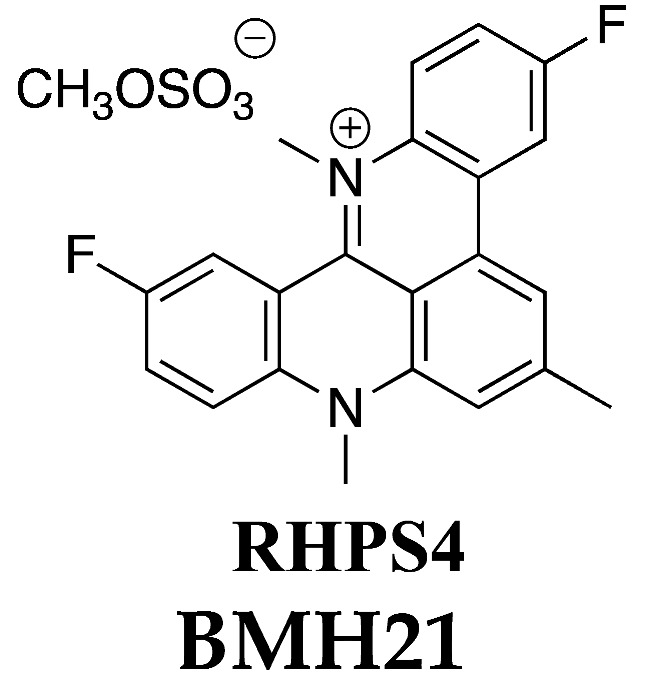	-	-	11.67 ± 1.53
**BMH21**	-	-	0.45 ± 0.02
**1a**	-H	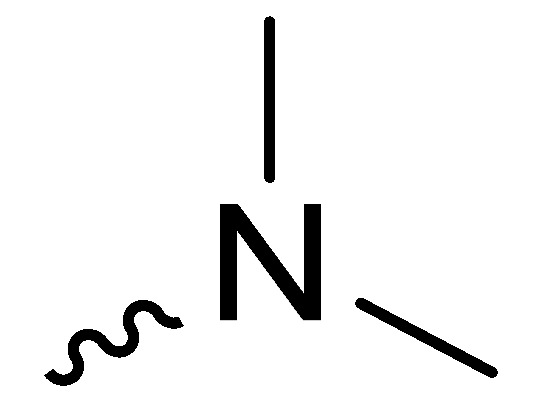	>100
**1b**	-OCH_3_	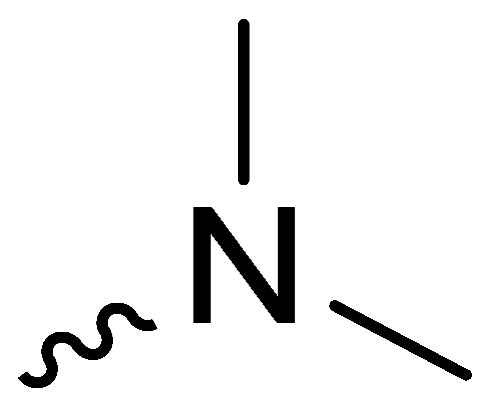	>100
**1c**	-H	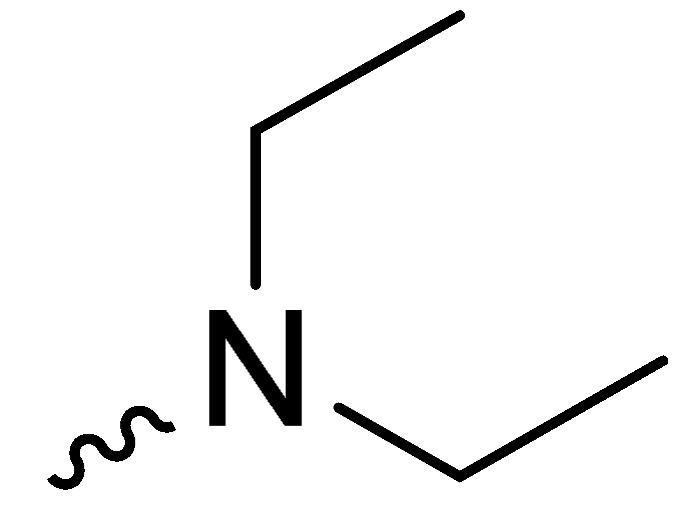	34.00 ± 1.00
**1d**	-OCH_3_	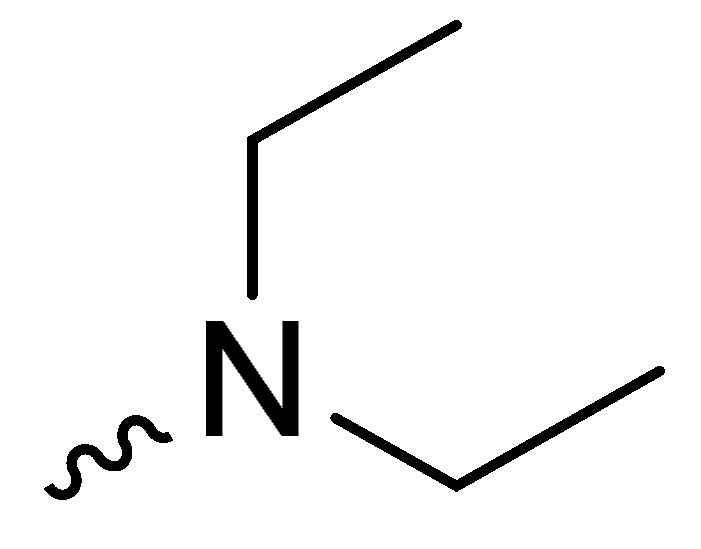	NT *
**1e**	-H	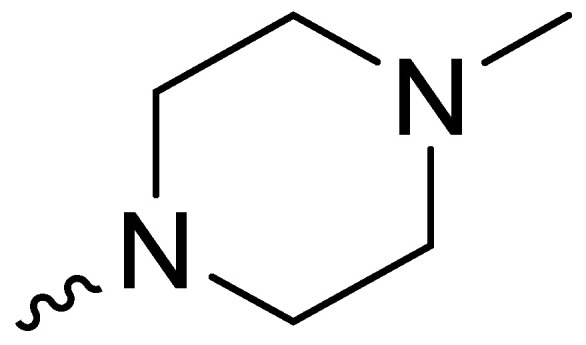	NT *
**1f**	-OCH_3_	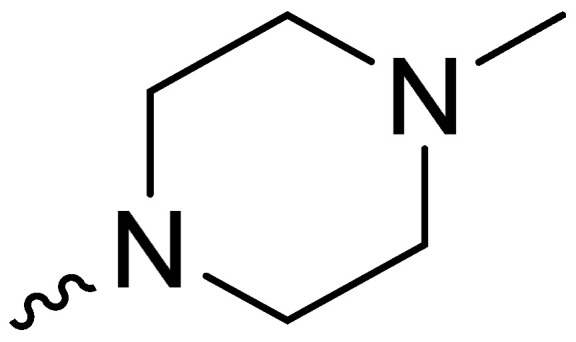	NT *
**1g**	-H	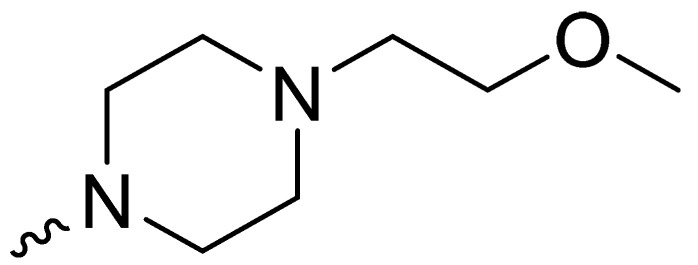	NT *
**1h**	-OCH_3_	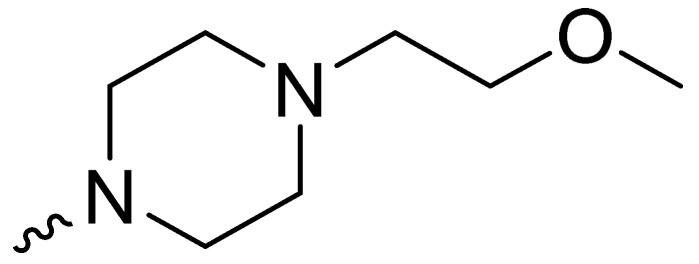	NT *
**1i**	-H	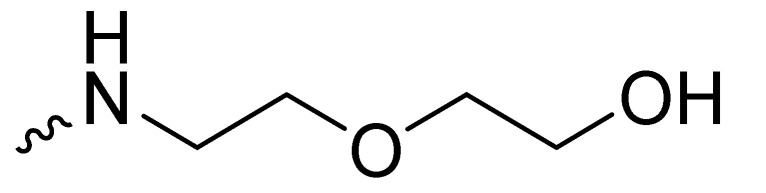	35.30 ± 2.08
**1j**	-OCH_3_	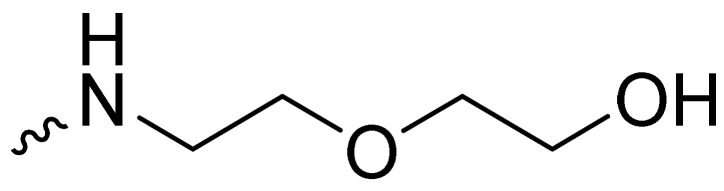	14.33 ± 2.08
**2a**	-H	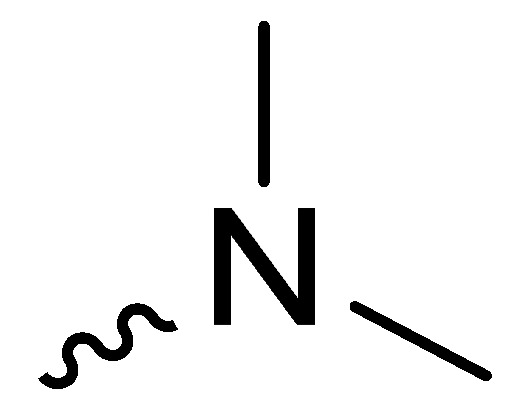	11.43 ± 1.50
**2b**	-OCH_3_	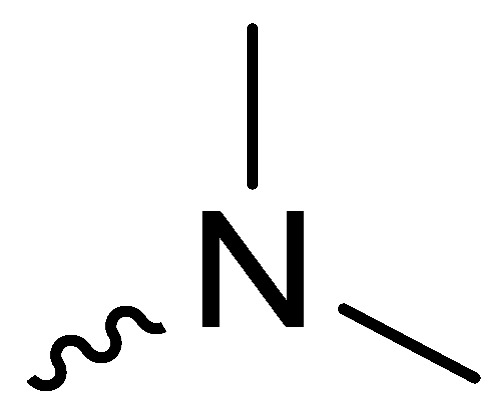	5.90 ± 0.26
**2c**	-H	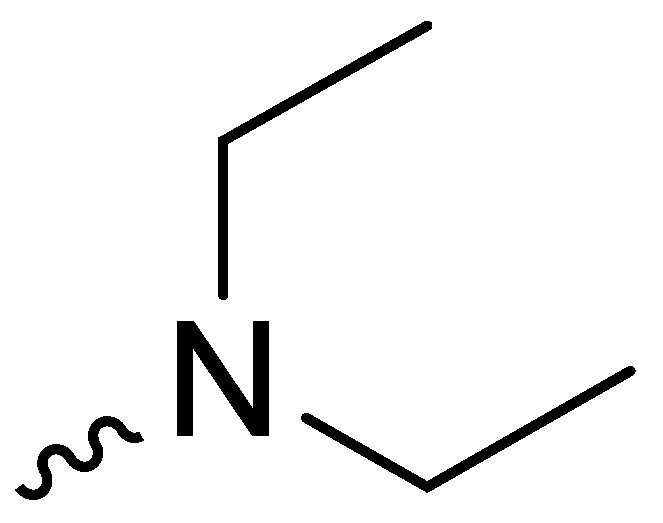	18.00 ± 4.58
**2d**	-OCH_3_	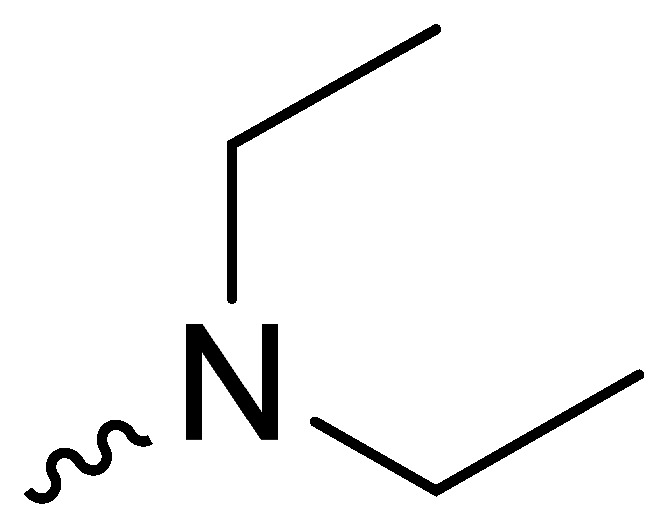	22.17 ± 1.75
**2e**	-H	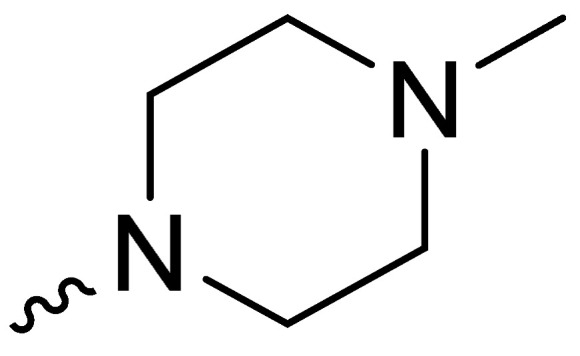	26.33 ± 2.08
**2f**	-OCH_3_	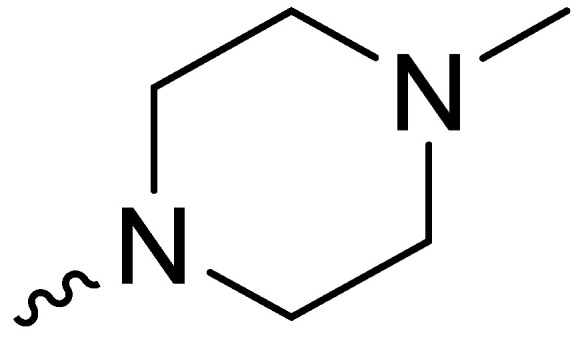	10.50 ± 0.50
**2g**	-H	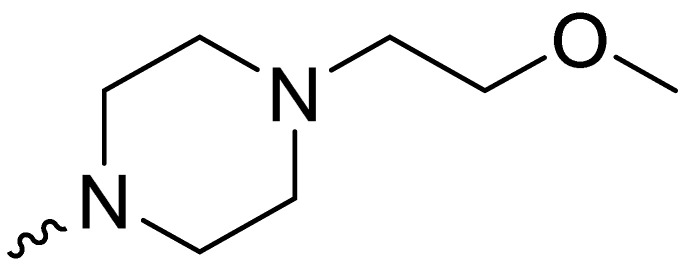	47.33 ± 24.09
**2h**	-OCH_3_	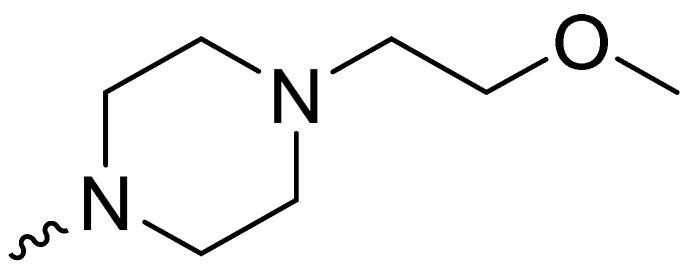	17.53 ± 1.28
**2i**	-H	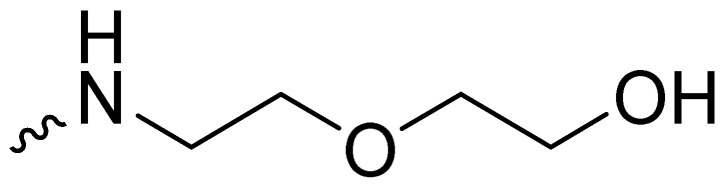	8.00 ± 1.00
**2j**	-OCH_3_	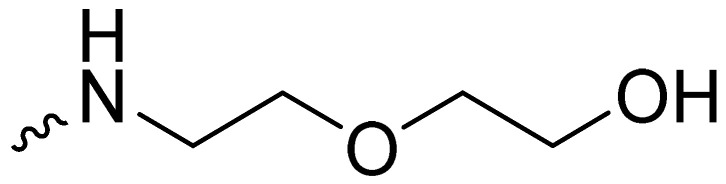	6.42 ± 0.24

^a^ Twenty-four hours after seeding, U2OS cells were exposed for 72 h to the compounds and cytotoxicity was measured using the MTS assay. The IC_50_ is defined as the drug concentration causing 50% cell growth inhibition, as determined by the dose-response curves. Data represent mean values ± SD of three independent experiments. * Not Tested due to the insolubility of each compound in DMSO.

**Table 2:** Selected ^1^H NMR chemical shift values for the complex of **2i** with Pu22-T14T23 G-quadruplex ^a,b^.

	H1/H2/Me	Δδ ^c^	H6/H8	Δδ ^c^	H1′
T4	1.57	−0.09	7.09	−0.12	5.72
G5	-	-	-	-	-
A6	n.d.	-	7.70	**+0.10**	n.d.
G7	11.36	**−0.38**	7.83	−0.19	6.08
G8	10.98	−0.24	7.68	−0.04	6.31
G9	10.56	−0.04	7.78	+0.03	6.27
T10	2.00	0.00	7.87	+0.06	6.53
G11	11.14	**−0.56**	7.83	−0.16	6.02
G12	11.14	**−0.35**	7.73	−0.17	6.10
G13	10.67	**−0.38**	7.74	−0.10	6.41
T14	1.95	+0.03	7.68	−0.03	6.25
A15	8.36	−0.01	8.56	+0.04	6.66
G16	11.48	**−0.42**	7.97	−0.14	6.31
G17	11.04	−0.20	7.66	−0.14	6.10
G18	10.69	**−0.32**	7.80	+0.01	6.42
T19	2.00	0.00	7.88	+0.02	6.53
G20	11.19	−0.09	8.03	+0.14	6.00
G21	11.19	−0.16	7.81	−0.10	6.02
G22	10.76	**−0.27**	7.81	+0.20	6.02
T23	1.80	**+0.32**	7.09	−0.05	5.62
A24	7.43	**+0.35**	-	-	-
A25	7.47	**+0.10**	7.57	+0.08	5.90

^a^ Measured at 25 °C in H_2_O/D_2_O (9:1), 70 mM KCl, 25 mM potassium phosphate buffer (pH = 6.9), and R = 4.0. ^b^ Measured in ppm from external DSS. ^c^ Δδ = δ_bound_ − δ_free_.

**Table 3:**
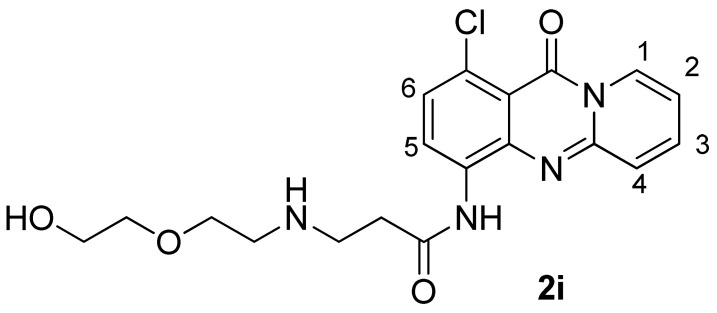
Chemical shift values of **2i**, and **2i** in complex with Pu22-T14T23 ^a^.

2i		Complex 2i/Pu22-T14T23	Δδ ^b^
H1	8.65	8.23	−0.42
H2	7.13	6.62	−0.51
H3	7.78	7.26	−0.52
H4	7.57	6.91	−0.66
H5	8.23	7.70	−0.53
H6	7.35	6.66	−0.69
CH_2_CO	2.57	2.70	+0.13
CH_2_NH	3.11	n.d.	-
CH_2_NH	3.39	n.d.	-
CH_2_O	3.54	3.60	+0.06
CH_2_O	3.65	3.78	+0.13
CH_2_OH	3.82	3.88	+0.06

^a^ Measured at 25 °C in H_2_O/D_2_O (9:1), 70 mM KCl, 25 mM potassium phosphate buffer (pH = 6.9), and R = 4.0. ^b^ Δδ = δ_bound_ − δ_free_. The reported H numbering is conveniently used only for NMR studies.

**Table 4:** Intermolecular NOE in the complex of **2i** with Pu22-T14T23 ^a^.

2i		Pu22-T14T23
	5′-binding site	
H2, H3, H4		H8G7
H5		NHG11
H5, H6		H8G16
H3		H1′G16
H5		H1′G16
	3′-binding site	
H4		H1′G9
H4		H8G22
H1		H1′G22
H1, H2, H3, H4		NHG18
H4		NHG13
H3		NHG22

^a^ Measured at 25 °C in H_2_O/D_2_O (9:1), 70 mM KCl, 25 mM potassium phosphate buffer (pH = 6.9), and R = 4.0.
